# Supplementary material for: Small-scale integrated farming systems can abate continental-scale nutrient leakage
Source: PLoS Biol. 2021 Jun 3;19(6):e3001264. doi: 10.1371/journal.pbio.3001264 (PMC8174726; doi:10.1371/journal.pbio.3001264)
Supplement: S1 Text — Supplementary information, including supplementary sections A–N, Eqs S1–S32, Tables A and B, and Figs A–F. (PDF) [file pbio.3001264.s002.pdf]

Supplementary Information for *Small-scale integrated farming systems can abate continental-scale nutrient leakage*

Gidon Eshel, geshel@gmail.com.

May 6, 2021

Contents

A The governing equations 2

A1 The manure partitioining paramter γ 4

B Solution strategy and approach 7

B1 Parameterizing nitrogen retention rates $\rho_{v,f}$ 14

B2 The vegetal yield factor ξ 16

B3 Sensitivity of total system productivity on the parameterized nitrogen retention rates $\rho_{v,f}$ 18

C From the individual farm results to the total cropland area 20

D The envisioned cattle diet 21

E Herd structure **22**

F Beef production **22**

G Estimating feed intake by the cattle herd **24**

H Estimating d, atmospheric nitrogen deposition rate **24**

I Estimating f_v, nitrogen fixing rates in the vegetal operation **25**

J Estimating f_f, rates of nitrogen fixation in the fodder operation **25**

K Estimating α, production rate of plant available manure nitrogen by a one cow "herd" **26**

L Estimating β, cattle needs for forage nitrogen **27**

M Evaluating and independently checking $\alpha\beta$ **28**

N Sensitivity of the results to the assumed yield penalty **29**

A The governing equations

A single operation (individual "unit" farm) comprises three parts (subunits): (1) vegetal human food production on A_v ha; (2) cattle fodder production on A_f ha; and (3) an intensive core cattle operation whose areal extent is negligible relative to A_v or A_f (here and throughout, v and f subscripts denote the vegetal and fodder production systems respectively).

The governing nitrogen balance equations for the vegetal, forage, and core cattle sub-units are

$$
\begin{aligned}
y_v &= \rho_v \left(d + f_v + \gamma \; \alpha \, n_m A_v^{-1}\right) && \text{(S1)} \\
y_f &= \rho_f \left(d + f_f + (1-\gamma) \, \alpha \, n_m A_f^{-1}\right) && \text{(S2)} \\
n_m &= \beta A_f y_f . && \text{(S3)}
\end{aligned}
$$

The left hand sides $y_{v,f}$ denote nitrogen yields—rates of nitrogen removal by harvest of the useful outputs—in kg N ha^{-1} y^{-1}, and d denotes atmospheric nitrogen deposition rates, also in kg N ha^{-1} y^{-1}. We assume d is spatially invariant on the small spatial scales of individual farms, holding over both A_v and A_f.

Rates of nitrogen fixation by root system symbionts in kg N ha^{-1} y^{-1} in the vegetal and forage operations are denoted $f_{v,f}$. On the right hand sides, $d + f_{v,f}$ are therefore the natural nitrogen inputs—atmospheric deposition and symbiotic fixation—into the vegetal and forage plots in kg N ha^{-1} y^{-1}. The rightmost right hand terms denote nitrogen addition by manure from the cattle herd, whose size is fully characterized by the number of mother cows n_m, as discussed in section E below. This herd produces αn_m kg of plant available manure nitrogen annually, where α is the annual production of plant available manure nitrogen by one cow and her associated animals (see section E for the precise definition of those associated animals). Of these αn_m kg plant available manure nitrogen y^{-1} the herd produces, in general a fraction $0 \leq \gamma \leq 1$ is allocated to the vegetal operation, while the remainder $1 - \gamma$ is directed to the forage operation, enhancing its production beyond the level permitted by natural nitrogen sources $d + f_f$.

In Eq. S3, β expresses cattle needs for forage nitrogen in (cow $\cdot$ y) (kg forage N)$^{-1}$. In Eq. S3, it multiplies total forage nitrogen output $A_f y_f$ to yield an expression for the herd

size n_m. More broadly, it plays a key role in the system's overall nitrogen cycling efficiency and productivity, as discussed in later sections.

The $\rho_{v,f}$ factors denote nitrogen retention rates. They represent the fact that nitrogen inputs (right hand sides of Eqs. S1 and S2) are imperfectly retained, because some fraction—$(1 - \rho_v)$ and $(1 - \rho_f)$ for the vegetal and forage subunits respectively—is lost by leaching into the environment as solutes in surface or shallow below surface flows [1]. While they have the appearance of specified paramters, here $\rho_{v,f}$ are treated as unknowns and are solved for. Because they are fractions, they fall inside [0,1], but some further of observational refinements are possible. An upper bound is offered by pristine forest environment. For example, N retention rates in Hubbard Brook approache but are just shy of 100% [3–5]. A lower bound is offered by modern intensive row crop agriculture, whose utilization efficiencies are as low as 50% [2, 3]. This is of course a key element of the problem with modern agriculture the nitrogen sparing agriculture (NSA) and similarly "sustainable" alternatives strive to rectify, so $\rho_{v,f} \approx [0.7, 0.95]$ are reasonable to expect in the current setting.

A1 The manure partitioining paramter γ

As written earliier, γ appears as a state variable. In fact, there is a straiightforward way to obtain a single optimal value for γ.

To show this, let's derive an expression for the productivity

$$\mathcal{P} := A_v y_v + \eta n_m \tag{S4}$$

of the system as a function of γ. $\mathcal{P}$ sums the system's two key outputs, vegetal food and beef, where $\eta \approx 10 - 12$ kg edible beef N the full herd produces annually per mother cow.

We assemble the building blocks for $\mathcal{P}$ by first recasting the equation for y_v to incorporate information about beef production as well. Using $n_m = \beta A_f y_f$, the yield equations become

$$y_v = \rho_v \left(d + f_v + \alpha\beta\gamma \frac{A_f}{A_v} y_f \right) \tag{S5}$$

$$y_f = \rho_f \left[d + f_f + \alpha\beta(1-\gamma) y_f \right]. \tag{S6}$$

The latter equation becomes

$$y_f - \rho_f \alpha\beta(1-\gamma) y_f = \rho_f (d + f_f) \quad \rightarrow \quad y_f = \frac{\rho_f (d + f_f)}{1 - \rho_f \alpha\beta(1-\gamma)}. \tag{S7}$$

Substituting this into the y_v equation finally yields

$$A_v y_v = A_v \rho_v (d + f_v) + \alpha\beta\rho_v\rho_f (d + f_f) A_f \left[\frac{\gamma}{1 - \rho_f \alpha\beta(1-\gamma)} \right]. \tag{S8}$$

The first of two additive terms in $\mathcal{P}$, the above takes note of the impact of γ on vegetal productivity by addressing both the rise of y_v with rising γ and the corresponding decline in manure supply due to declining y_f with rising γ.

The second $\mathcal{P}$ term, addressing beef supply, is

$$\eta n_m = \eta\beta A_f y_f = \eta\beta A_f \frac{\rho_f (d + f_f)}{1 - \rho_f \alpha\beta(1-\gamma)} \tag{S9}$$

and thus

$$\mathcal{P} = A_v \rho_v (d + f_v) + \frac{\alpha\beta\rho_v\rho_f (d + f_f) A_f \gamma + \eta\beta A_f \rho_f (d + f_f)}{1 - \rho_f \alpha\beta(1-\gamma)} \tag{S10}$$

or

$$\mathcal{P} = A_v \rho_v (d + f_v) + \frac{\beta A_f \rho_f (d + f_f) \left[\alpha\rho_v\gamma + \eta \right]}{1 - \rho_f \alpha\beta(1-\gamma)} \tag{S11}$$

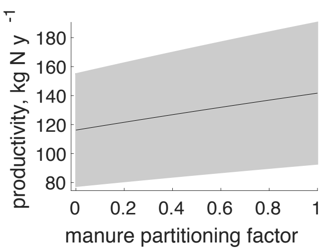

Figure A: Dependence of total system productivity $\mathcal{P}$ on the manure partitioning coefficient γ (where $\gamma = 0$ and $\gamma = 1$ mean the vegetal plot gets none and all the manure the cattle herd produces). The solid curve and shading present the most likely value and spread about it. Possible yet improbable more extreme values outside the shaded region are not shown.

shown in Fig. A. Clearly $\partial y_v/\partial\gamma > 0$ throughout, i.e., the system's productivity rises with γ all the way to $\gamma = 1$ which is thus the optimal value.

With the optimal $\gamma = 1$, the governing equations reduce to

$$y_v = \rho_v \left(d + f_v + \frac{\alpha\ n_m}{A_v} \right) \tag{S12}$$

$$y_f = \rho_f \left(d + f_f \right) \tag{S13}$$

$$n_m = \beta A_f y_f \tag{S14}$$

or

$$y_v = \rho_v \left(d + f_v \right) + \alpha\beta\rho_v\rho_f \left(\frac{A_f}{A_v} \right) \left(d + f_f \right). \tag{S15}$$

Because α, β and $\rho_{v,f}$ are all fractions by definition, as is (as will be shwon shotly) A_f/A_v, the coefficient multiplying the N input into the forage subunit is very small, which highlights the relative inefficiency with which forage N inputs transform into vegetal output. Excluding ρ_v, the factor common to nitrogen input in the vegetwal and forage subunits, $\alpha\beta\rho_f\left(\frac{A_f}{A_v}\right)$ ranges over $\approx$ 0.03–0.2, and is typically (in most Monte Carlo realizations) $\mathcal{O}(10^{-2} - 10^{-1})$. This means that because of the inefficient transformation of forage nitrogen into beef, every 10 nitrogen units available in the forage subunit are jointly roughly equivalent to only 1 unit

in the vegetal subunit, a distinct disadvantage that explains the optimality of $\gamma = 1$ despite the fact that the dominant N source, symbiotic fixation, is more often than not larger in the forage subunit (i.e., $f_f > f_v$).

B Solution strategy and approach

We will still need to manipulate Eq. S15 a bit to make it practically useable for the current situation. The following introduction of the general solution strategy will explain why and how.

First, we choose specific $A_{f,v}$—the vegetal and forage areas in a single unit farm—and thus A_f/A_v. Recent years' U.S. total cropland used for crops has been [6] just under 140 million ha, of which corn, soy, sorghum, barley and oats [7] claimed about 41 million ha. Because on average feed use of those crops is about 45%, their feed related areal claims amount to approximately 18 million ha. Adding hay and haylage, which span about 23 million ha and are used exclusively for feed, feed crops claimed roughly 41 million ha. This is almost exactly 30% of the 140 million ha U.S. total cropland used for crops reported above. Today, therefore, cropland is partitioned among feed and other uses (e.g., food, biofuel) as approximately 30:70. In light of this, and because here cattle is primarily a manure source of secondary import, we set

$$\left.\begin{array}{rcll} A_v &=& 1\text{ ha} & \\ A_f/A_v &=& 3/7 \quad \rightarrow & A_f \approx 0.43\text{ ha} \end{array}\right\} A_t = \frac{10}{7}\text{ ha} \approx 1.43\text{ ha} \qquad \text{(S16)}$$

where A_t is the total area of the envisioned unit farm.

We solve the system in a Monte Carlo framework in which specific realizations of

$\{d, f_{v,f}, \alpha, \beta\}$ are randomly drawn from corresponding distributions derived from robust observations of lower and upper bounds of these paramters. Because α and β depend on cattle diet, we consider three such diets (see Table A) whose quality ranges widely enough to collectively fully bracket the range of diets that can be plausibly expected to actually characterize a realistic deployment of the envisioned scenarios.

With the above paramteres determined or specified for a given realization, the solution sequence for a given Monte Carlo realization is as follows.

1. Draw random values for $\{d, f_{v,f}, \alpha, \beta\}$ from the uniform distributions characterized by the extreme values derived in sections H, I, J, K and L respectively.

2. Choose 12 random non-N-fixing plant items for the specific Monte Caarlo realization.

3. Choose 3 random N-fixing plant items for the specific Monte Caarlo realization.

4. The above two determine the basic composition of (item representation in) the realization-specific vegetal mass and N yield vectors $\mathbf{x}_v$ and $\mathbf{y}_v$ in g and g N y^{-1} respectively.

5. Obtain deposition plus symbiotic fixation N inputs into the vegetal and forage subunits, $d + f_f$ and $d + f_v$.

6. Use these N inputs to calculate $\rho_{v,f}$ as described in section B1. For the vegetal subunit, the N input into the ρ_v function would ideally also include the manure addition. But this would require an iterative solution procedure (because the mass of available manure N is itself a function of n_m and y_f which both depend on ρ_v). We have used such iterative solution early on in this work, at great computational burden, but eventually abandoned this because of the minimal impact on the results. It is easy

to see why this impact is small; the full input is $\rho_v\,(d + f_v) + \alpha\beta\rho_v\rho_f\left(\frac{A_f}{A_v}\right)(d + f_f)$ (Eq. S15), whose manure representing second term is roughly the first term times $\alpha\beta\rho_f A_f A_v^{-1} \ll 1$ (because d is uniform and the ranges of f_v and f_f are quite similar). To leading order, therefore, the nitrogen input into the vegetal plot is $d + f_v$.

7. Use Eq. S15 to obtain the *full* nitrogen input into the vegetal subunit, $\rho_v\,(d + f_v) + \alpha\beta\rho_v\rho_f\left(\frac{A_f}{A_v}\right)(d + f_f)$.

8. Use this N_{in} to calculate the vegetal yield factor ξ (section B2).

9. Obtain the final (properly scaled) scalar vegetal N yield, $y_v = \xi\bar{\mathbf{y}}_v$, where the overbar denotes averainge over $\mathbf{y}_v$'s 15 elements, not ensemble mean.

10. Obtain the forage N yield, $y_f = \rho_f(d + f_f)$.

11. Obtain the herd size, $n_m = \beta A_f y_f$.

12. Obtain beef production, n_m times the 125–155 kg edible beef produced per year per mother cow in the herd, given in the bottom of Table B.

13. Use the mass yield vector $\mathbf{x}_v$ to obtain the nutrtional delivery by and environmental savings associated with the vegetal portion of this MC-specific diet.

14. Add the beef nutrtional delivery and environmental savings to the above plant-based nutrtional delivery.

All steps above that have not yet been described unambiguously are described in subsequent sections.

Overall, we consider 61 plant items whose full nutritional and environmental information we have previously published and used [8], introduced by name and nitrogen yields in Fig. B.

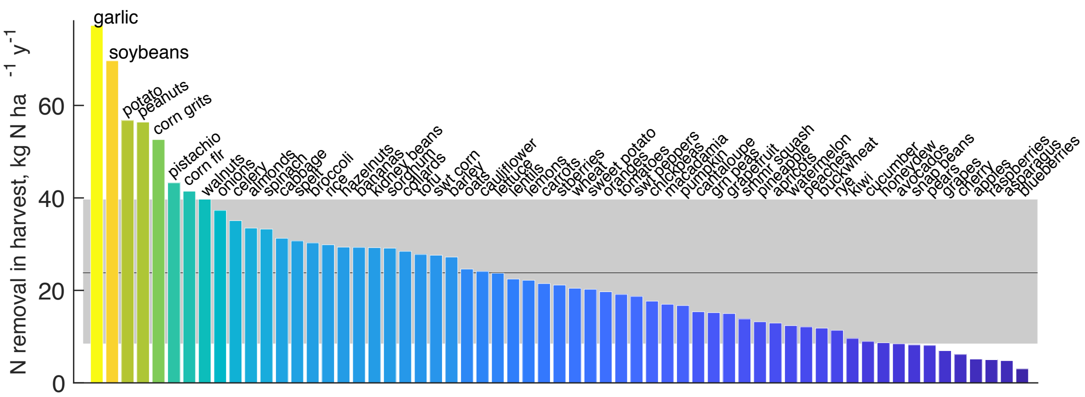

Figure B: Nitrogen removal rate by harvest of key fruits and vegetables. Based on previously published data [8]. The shading spans the middle 50% of the distribution of N removal rates.

Of those, 7 are nitrogen fixing vegetal items (e.g., soy, peanuts). In each Monte Carlo realization, we choose randomly 3 nitrogen fixing vegetal items and 12 additional ones, and partition the $A_v = 1$ ha of the vegetal plot equally among the 15 items. The $1/15 \approx 0.07$ ha allocated for each crop yields a certain mass output that takes simultaneous note of the item's known current yield [8] and the depositional plus fixation-derived nitrogen availability in the vegetal subunit in the specific Monte Carlo realization, $d + f_v$, as introduced above and discussed in section B1.

Because the overall system we consider is widely distributed, small scale and unlikely to enjoy economy of scale characteristic of today's conventional operations, we demote the yields by 15% and assume they are 85% of today's. This value is guided by characteristic long term yield ratios found in meta-analytic comparisons of conventional and organic agriculture [9, 10], the latter standing here as a defensible if imperfect proxy for small scale, environmentally mindful operations whose very structure is premised on forgoing economy of scale benefits. To be clear, in departure from earlier work, the plant item land allocation is not subject to optimization. In making this choice, we accept allocation suboptimality as a reasonable price to pay for the assurance that our model system produces realistic diets

rather than optimized diets that may differ too starkly from typical culinary preferences to be widely availed by the populace.

Using official nutritional composition of the plant items [11], these demoted total mass yields can be readily translated into the annual delivery of protein, or B_{12}, or any other micro or macro nutrient for which USDA information [11] exists. Combined, these different yields fully characterize the output diet a specific randomly chosen $A_t = 10/7$ ha farm being considered delivers, both from a culinary standpoint—x kg cauliflower or y kg garlic—and nutritionally, e.g., the farm delivers x kg fat or y μg α carotene annually. Together, these yield types completely quantitatively describe the plant based component of the diet persons eating the output of the considered farm can enjoy.

The same, albeit following a different logical path, holds for the beef based part of the diet. Once n_m is gotten (by solving Eq. S14), we use the herd structure (see section E and Table B) to calculate the annual beef mass the $A_v \approx 0.43$ ha part of the operation yields. As with the plant based part of the diet, we also translate this beef mass yield (using the nutritional attributes [11] of extra lean grass fed beef) into the nutritional consequences of the farm's beef output in terms of the same set of micro or macro nutrients [11]. The sum of nutrients the vegetal and beef parts of the randomized configuration yields is the total dietary quality of the combined farm's output.

Let's symbolically summarize the above as follows. Let $\mathbf{x} \in \mathbb{R}^{62\times 1}$ be the mass output vector. For a given Monte Carlo realization, $\mathbf{x}_a \subset \mathbf{x}$, where

$$\mathbf{x}_a := \left[\, \mathbf{x}_v^T \; x_b \, \right]^T \in \mathbb{R}^{16\times 1} \tag{S17}$$

has 16 nonzero elements: 3 correspond to the 3 randomly selected nitrogen fixing plant items, 12 correspond to the 12 randomly selected other (non nitrogen fixing) plant items,

and a final one—x_b—corresponding to beef. The numerical values of these nonzero elements reflect the expected mass yield. For example, if one of the randomly selected plant items in a particular Monte Carlo realization is onion, the corresponding $\mathbf{x}$ element will be

$$x_{\text{onion}} = \frac{1}{15}\,[\text{ha}] \times 0.85 \times 23810 \left[\frac{\text{kg onion}}{\text{ha} \cdot \text{y}}\right] \approx 1587 \left[\frac{\text{kg onion}}{\text{y}}\right],$$

where 1/15 ha is the equal area in A_v allotted for each of the 15 participating plant items, 0.85 is the yield demotion factor, and 23810 kg onion (ha y)$^{-1}$ happens to be the current national mean onion yield [8, 12].

The $\mathbf{x}$ element corresponding to beef is a linear function of the solved for n_m (Eq. S14), as discussed in details in section F and the bottom of table B. For now, we can simply state the bottom line; for every mother cow (i.e., for every $n_m = 1$ increment), 125–155 kg beef is produced annually on the $A_f \approx 0.43$ ha foragew sybunit, where the specific value within this range depends on the specific cattle diet quality chosen, as explained further in sections D–F.

Next, let's introduce the two data matrices $\mathbf{N} \in \mathbb{R}^{62\times40}$ and $\mathbf{E} \in \mathbb{R}^{62\times2}$. Element ij of $\mathbf{N}$, N_{ij}, holds the jth nutrient content in the ith food item, while element ik of $\mathbf{E}$, E_{ik}, holds the kth environmental cost of the ith food item, where $k \in [1, 2]$ can be either nitrogen fertilizer use or greenhouse gas emissions. For example, N_{ij} is 1.5 kcal (g garlic)$^{-1}$ when i corresponds to garlic and j to dietary calories, and E_{ik} is 1.1 g $CO_{2\text{eq}}$ (g spinach)$^{-1}$ when i corresponds to spinach and k to greenhouse gas emissions.

With $\mathbf{x}$, $\mathbf{N}$ and $\mathbf{E}$ thus defined,

$$\frac{\mathbf{N}^T\mathbf{x}}{A_t} = \mathbf{d} \in \mathbb{R}^{40\times1} \tag{S18}$$

$$\frac{\mathbf{E}^T\mathbf{x}}{A_t} = \mathbf{e} \in \mathbb{R}^{2\times1} \tag{S19}$$

hold the nutritional and environmental consequences of allocating $A_v + A_f \approx 1.43$ ha of high quality land to the envisioned production system. The elements of **d** are the nutritional yields, e.g., d_1 kcal ha^{-1} y^{-1} and d_2 g protein ha^{-1} y^{-1} quantify the caloric and protein yield per ha the system delivers with the $A_v = 1$ ha equally distributed among the 15 randomly chosen plant items, and with the $A_f \approx 0.43$ ha beef operation producing an annual beef mass that reflects the specified quality of the forage cattle diet.

The elements of **e** are the *averted* fertilizer use and greenhouse gas emissions the full (plant and beef) output diet would have required had it been produced using today's methods. The two elements of **e** are (see Eq. S19) the sums of what is actually produced—x_i in kg product per ha per year—times the nitrogen fertilizer or greenhouse gas emission costs per unit mass the production of that specific product requires today using conventional, mostly industrial agriculture. So the term "averted"—while identically correct for fertilizer, because none is used in the envisioned NSA model—is not fully justified for greenhouse gas emissions. That is, the lion's share of emissions directly related to fossil fuel combustion (as opposed to land use and land use changes) is incurred by conventional agriculture in the production of agrochemicals (herbicides, pesticides, fungicides and fertilizer). Because these chemicals are not used in the NSA model, their associated emissions are indeed fully averted, representing unambiguous savings. But a small amount, typically 10-15% and rarely as high as 25% [13, 14], related to field operations is still required in the NSA model. Referring to the elements of **e** as "averted" is thus fully justified for e_1, corresponding to nitrogen fertilizer, but only 75–90% justified for emission-related e_2.

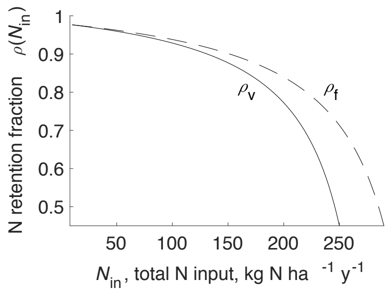

Figure C: Dependence of parameterized nitrogen retention in the vegetal and forge subunits (ρ_v and ρ_f in dashed and solid respectively) on total (deposition plus fixation, $d+f_{v,f}$) nitrogen input.

B1 Parameterizing nitrogen retention rates $\rho_{v,f}$

Nitrogen retention rates are bracketed by two empirical endmembers. The first is the above-quoted 50% characterizing modern intensive row crop agriculture [2, 3]. The second involved N retention rates in such well observed pristine natural ecosystems as Hubbard Brook, whose N utilization approaches 100% [3–5] despite relatively rapid surface and near surface hydrology induced by steep topography. Retention rates thus range roughly inside 50%–100%, with the lower and upper end characteristic of high productivity industrial agriculture and minimally perturbed natural ecosystems respectively, and an expected nonlinear variation between those two extremes for perturbed yet more sustainable agroecosystems this paper addresses.

With this background, we proceed to parameterize $\rho_{v,f}$ as Michaelis–Menten-like processes,

$$\rho_{v,f} = \rho^{min} + \frac{a_1 N_{in,v/f}}{\tilde{N}_{in,v/f} + N_{in,v/f}} \tag{S20}$$

where the subunit-specific varying input is $N_{in,v/f} := N_{in}^{min} - \left(f + f_{v/f}\right)$.

$$\text{Above,}\;\; a_{1,v/f} = \left(\rho^{max} - \rho^{min}\right)\left(\tilde{N}_{in,v/f}/N_{in,v/f}^{max} + 1\right), \tag{S21}$$

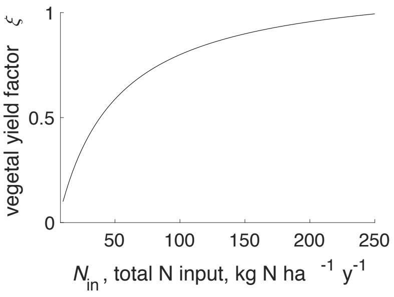

Figure D: Dependence of ξ, the vegetal yield demotion factor, on N_{in} (Eqs. S15 and S26), the system-wide nitrogen flux that is productively available (after accounting for all losses and inefficiencies) for vegetal production. This is the factor by which today's observed vegetal yields are demoted based on nitrogen availability. For exmaple when N_{in} attains the lowest value possible in our Monte Carlo implementation (left vertical gray line at $N_{in} = 44$ kg N ha^{-1} y^{-1}), today's vegetal nitrogen yields of all plant item that were randomly selected or a particular Monte Carlo realization—$\{y_i\}$, $i = [1, 15]$—are replaced by $0.3\,\{y_i\}$ (where the 0.3 value is shown aws the height of the vertical gray line at $N_{in} = 44$ kg N ha^{-1} y^{-1}).

$\rho^{min} = 0.45$ and $\rho^{max} = 0.98$, the Hubbard Brook N utilization [3–5], $N_{in}^{min} = 10$ kg N ha^{-1} y^{-1}, $N_{in,v/f}^{max} = 250$ and 290 kg N ha^{-1} y^{-1}, and

$$\tilde{N}_{in,v/f} = \left(N_{in,v/f}^{max} - N_{in}^{min}\right)/5 = 48 \text{ and } 56 \text{ kg N ha}^{-1}\text{ y}^{-1} \tag{S22}$$

for the v and f subunits respectively.

The dependence of $\rho_{v,f}$ thus parameterized on N input is shown in Fig. C. The varying input into this paramterization is $d + f_{v,f}$ for the vegetal and forage subunits, the sum of per ha atmospheric deposition and symbiotic fixation. We examine the sensitivity of the results to this nitrogen retention paramterization in section B3.

B2 The vegetal yield factor ξ

Recall that the vegetal nitrogen yield of a given Monte Carlo realization is $y_v = \xi \bar{\mathbf{y}}_v$ where $\mathbf{y}_v \in \mathbb{R}^{15}$ is the realization-specific plant yield 15-vector, whose mean over all elements (*not* ensemble mean!) is $\bar{\mathbf{y}}_v$. The reaseon this mean is used is the equql area allocation; since the 15 crops participating in a given Monte Carlo realiization are uniformly spatially distributed, with each occupying 1/15th ha, the mean yield per ha is simply the arithmetic mean of all $\mathbf{y}_v$'s elements, $\bar{\mathbf{y}}_v$.

The need for the $y_v = \xi \bar{\mathbf{y}}_v$ representation arises because the governing equations address the full allocated area, not individual crops within that area. That is, collecting productively available nitrogen from the vegetal and forage subunits, the right hand side of

$$y_v = N_{in} = \rho_v (d + f_v) + \alpha\beta\rho_v\rho_f \left(\frac{A_f}{A_v}\right)(d + f_f). \tag{S23}$$

(Eq. S15) only applies to the vegetal subunit as a whole, sheding no light on the yield of individual crops therein.

Overcoming this inherent ambiguity of the highly simplified model used here is the rationale for the

$$y_v = \xi \bar{\mathbf{y}}_v \tag{S24}$$

representation. Its action is readily underdtood by appeal to Fig. D. The vector of today's yields, $\mathbf{y}_v$, plays two roles. First, it provides structure. For example, the element corresponding to garlic is 15 times the element corresponding to apples because garlic yields 15 times the nitrogen per ha per year apples do; $y_v^{\text{garlic}} = 15 y_v^{\text{apples}}$. Second, $\mathbf{y}_v$ provides the numerical upper bounds; The most garlic can yield is ≈ 66 kg nitrogen ha^{-1} y^{-1}, 85% of today's actual garlic nitrogen yield (because, recall, even prior to the possible demotion by ξ,

we reduce yields across the board by 15% to account for the envisioned operation's reduced efficiency).

With these two roles, ξ demotes each of the yields in $\mathbf{y}_v$ according to nitrogen availability to the vegetal subunit. This is guided by the assumption that today's yields closely track complete or near-complete elimination of the nitrogen limitation on growth and are thus proper upper bounds with respect to nitrogen availability. In individual Monte Carlo realizations, these levels of nitrogen supplies are rarely attained; in most cases, depending on the vagaries of $f_{v,f}$ and d randomization, less nitrogen is available, and the expected yields are correspondingly smaller than 85% of today's. This demotion is achieved by the $\xi(N_{in}) \leq 1$ factor, which is presented grahically in Fig. D.

The analytic form of $\xi(N_{in})$ is

$$\xi(N_{in}) = \xi_{min} + \frac{b_1 N_{in}}{\tilde{N}_{in} + N_{in}} \tag{S25}$$

where the subscript v is suppressed with no added ambiguity, with the varying input

$$N_{in} := \left[\rho_v(d + f_v) + \frac{A_f}{A_v}(d + f_f)\alpha\beta\rho_v\rho_f \right] - N_{in}^{min} \tag{S26}$$

in which the non-essential brackets hold and thus clearly identify the total productively available nitrogen input into the vegetal subunit, the right hand side of Eq. S15.

$$\tilde{N}_{in} = \left(N_{in}^{max} - N_{in}^{min}\right)/5 \approx 40 \text{ kg N ha}^{-1} \text{ y}^{-1}, \tag{S27}$$

$N_{in}^{min} = 35$ kg N ha^{-1} y^{-1}, and $N_{in}^{max} = 230$ kg N ha^{-1} y^{-1}, as before, and

$$b_1 = \left(\xi^{max} - \xi^{min}\right)\left(\tilde{N}_{in}/N_{in}^{max} + 1\right), \tag{S28}$$

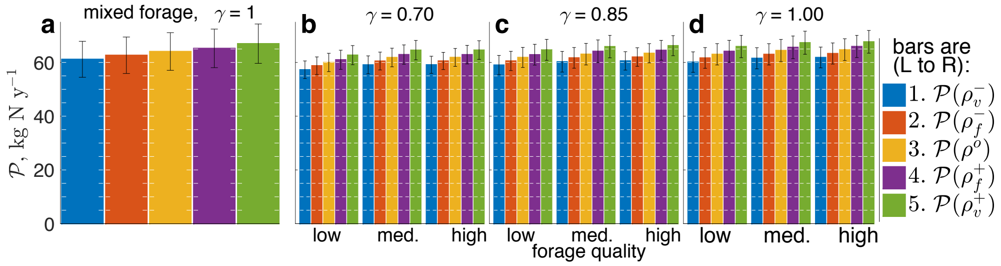

Figure E: Sensitivity of total systrm productivity $\mathcal{P}$ (Eq. S11) to uncertainty in the nitrogen retention paramterization ($\rho_{v,f}$ in the vegetal and forage subunits). The main result is in panel a, where all forage qualities are considered simultaneously and the manure partitioning is the optimal value, $\gamma = 1$. In all panels, the bar heights correspond to the full-farm productivity $\mathcal{P}$ in kg N y^{-1}. It is convenient to think of $\mathcal{P}(\rho_{v,f})$ as a function of two key inputs, ρ_v and ρ_f. The five color-coded bars in each group (see legend on right) correspond to evaluations of this function with inputs (from left to right) $\left\{\rho_v^{-5\%}, \rho_f\right\}$, $\left\{\rho_v, \rho_f^{-5\%}\right\}$, $\left\{\rho_v, \rho_f\right\}$, $\left\{\rho_v, \rho_f^{+5\%}\right\}$ and $\left\{\rho_v^{+5\%}, \rho_f\right\}$ respectively, where, e.g., $\rho_v^{\pm 5\%}$ denotes replacing ρ_v with $1.05\rho_v$ and $0.95\rho_v$ in the input arguments into $\mathcal{P}(\rho_{v,f})$, *all after perturbing simultaneously and mutually independently* a_1 *and* $\tilde{N}_{in,v/f}$. Spread whiskers are $\pm$ one standard error. In a they are derived from the 750-member population comprising 250 Monte Carlo realizations times 3 forage qualities, while in b–d it is only over the 250 Monte Carlo realizations.

with $\xi_{min} = 0.1$ and $\xi_{max} = 1$.

B3 Sensitivity of total system productivity on the parameterized nitrogen retention rates $\rho_{v,f}$

The $\rho_{v,f}$ paramterizations we employ (section B1 and Eq. S20) are preliminarily suspect for two reasons. First, their paramter values are ad hoc and do not reflect fundamental first principles. Second, while their analytic form is reasonable and familiar, it too is not firmly rooted in general observations or widely agreed-upon consensus.

Consequently, and because of the prominence in the governing equations of the paramterized $\rho_{v,f}$, we study the sensitivity of the solution to uncertainty inherent in this parameterization. A reasonable metric for evaluating the sensitivity is total system productivity $\mathcal{P}$ (Eq. S11).

We thus repeat the full calculation nine times (3 forage qualities times 3 γ values, 0.7, 0.85 and the default optimal 1), exploring the system productivity under multiple perturbations that simultaneously address both above potential limitations of the paramterization.

For each Monte Carlo realization, in each of the above nine combinations of specific forage quality and γ, in addition to the normal randomization of the 15 plant items and $\{\alpha, \beta, d, f_{v,f}\}$, we also (1) perturb $\pm 10\%$ parameters a_1 and $\tilde{N}_{in,v/f}$ (Eqs. S21 and S22) mutually independently; and (2) additionally perturb $\pm 10\%$ the resultant calculated $\rho_{v,f}$. These two perturbations reflect the two potential problems introduced above: uncertain paramter values, and potentially imperfect analytic form of the paramterization.

This procedure means five $\mathcal{P}$ estimates—each independently randomized as above—for each individual Monte Carlo realization, one in which neither $\rho_{v,f}$ are perturbed, and four in which either is perturbed individually (independently of the other) 5% up and then down (see caption of Fig. E). The statistics of these experiments are summarized in Fig. E, with further illuminating but non-essential details presented in panels b–d.

Reassuringly, the results of these calculations (Fig. E) show that the key model prediction—total system productivity $\mathcal{P}$—is only weakly dependent on the imperfectly parameterized nitrogen cycling efficiencies. By presenting small and insignificant differences among the 5 perturbation categories (the 5 colored bars in Fig. Ea and in each forage quality group in panels b–d), and by exhibiting spreads that are modest fractions of the reference values (the height of any spread whisker as a fraction of that of the corresponding bar), Fig. E shows perfectly acceptable sensitivities of the full-farm productivity $\mathcal{P}$ to the general analytic structure of and specific governing parameter values in the nitrogen cycling efficiency parameterization.

C From the individual farm results to the total cropland area

Recall that once an individual farm Monte Carlo solution is obtained, the resultant produced vegetal plus beef diet must be scaled up (multiplied) by a total area over which the considered NSA system can be reasonably expected to be deployed. This quantify the expected national outputs, and those can be divided by 330 million Americans to yield the national mean per capita diet. The averted environmental costs can be similarly scaled up to the total expected national environmental savings.

To estimate the required total cropland area, we use the mean 2007 and 2012 U.S. Agricultural Census cropland use values [6] (where cropland is the higher quality subset of the overall agricultural land category, and the two successive censuses are extremely close). Spatially, we include the USDA agricultural districts of the Northeast, Lake States, Corn Belt, Northern Plains, Appalachia, Southeast, and Delta States , but exclude the Southern Plains, Mountains, and Pacific (the latter encompassing the entire Pacific seaboard). Excluding the Pacific district—despite the bountiful precipitation most agricultural lands in Washington, Oregon and California west of the Cascades and the Coast Range enjoy—makes our nutritional outputs and environmental savings estimates conservative, by roughly the 3–4% (the portion half of the cropland in those three states constitute of our considered total).

With the above consideration, the included district span 105 million ha, which is the value we use for national upscaling.

Table A: Three representative diets based on NRC [15] data.

feed	Mcal kg^{-1} NE^d_m	NE^d_g	NE^d_l	% crude protein
— high quality forage —				
fresh wheat pasture	1.46	0.91	1.44	20
fresh young sudangrass	1.44	0.87	1.40	17
fresh ladino clover	1.40	0.85	1.38	25
**mean high quality**	**1.43**	**0.88**	**1.41**	**20**
— medium quality forage —				
young Timothy hay	1.16	0.55	1.16	11
midbloom alfalfa hay	1.14	0.51	1.14	17
grass hay	1.14	0.51	1.14	10
**mean med. quality**	**1.15**	**0.52**	**1.15**	**13**
— low quality forage —				
fresh sagebrush	0.98	0.24	0.96	13
prairie hay	0.98	0.24	0.96	7
mature alfalfa hay	0.98	0.24	0.96	13
**mean low quality**	**0.98**	**0.24**	**0.96**	**10**
— high quality industrial byproducts —				
distillers grain corn	2.26	1.52	2.13	28
whole cottonseeds	2.11	1.44	1.95	23
wheat millfeed	1.69	1.10	1.63	18
**mean byproducts**	**2.02**	**1.35**	**1.90**	**23**

D The envisioned cattle diet

In the current setting, the herd size is determined by manure production, which depends not only on the animal numbers and sizes already discussed but also on the composition of the rations and the amounts actually consumed. We consider separately high, medium, and low quality idealized forage diets, described in table A. Because the envisioned herd subsist exclusively on low-intensity, extensive fodder which typically comprises multiple species and various maturity levels, we consider the idealized forage based diets as endmembers whose combinations span most realistically encountered mixed fodder cattle diets.

E Herd structure

The specifics of the herd structure depend on the number and weight of all the various animals in the herd. As already mentioned, calving mother cows are the core of the beef operation, and their number n_m is the herd's defining variable, from which numbers of all other category members of the herd [16–18] follow. These numbers and their representative body weights [16–18] are fully detailed in table B, including the implicit assumption of 15% replacement rate for both genders [17, 18], and a death rate of 10% for growing animals.

F Beef production

The herd beef yields per one mother cow are as follows. Since we assume mature cow replacement rate of $0.15n_m$, each cow contributes $0.15 \times 600 = 90$ kg live weight per year of culled mature mother cows.

Bull replacement per mother cow yields $0.15 \times 0.04 \times 820 \approx 5$ kg live weight per year of culled bulls, which is minutely further raised by death of growing heifers and bulls in the replacement pipeline.

The main output beef stream is due to finishers, at an approximate rate of $0.7\times$ (450–600) kg live weight per year.

Adding the above contributions, total annual live weight beef production is about 520–540 kg per cow.

We assume[19] live to hot carcass losses of 39% (i.e., we assume a dressing percentage of 61), and subsequent losses of 49% [20], for a total supply chain mass retention of 30%, so that a

Table B: The characteristics of the cattle herd and their NRC-predicted [15] fodder intake, assuming 90% live delivery, 15% annual replacement of mature stock, and 10% death of growing animals. Within animal groups, top and bottom rows correspond to high and low diet quality respectively.

cattle type	num. per 100 cows	approximate body mass, kg: initial	approximate body mass, kg: final	days in phase per y	predicted mean feed intake, kg dry matter $(\text{head} \cdot \text{d})^{-1}$: high quality	med. quality	low quality	kg y^{-1} edible beef yield
mature cows	100	600	600	365	13.6	12.7	12.4	27
mature bulls	4	820	820	365	15.8	15.7	15.2	1
		35	430		5.4			
unweaned calves	86	35	280	220		3.9		
		35	165				2.6	
		430	600	100	9.4			124
fattened $\leq$2 y animals[a]	69	280	600	305		8.0		124
		165	450	510			5.8	93
		430	620	365	11.6			3
replacement heifers[b]	16	280	615	385		9.4		2
		165	570	695			7.0	2
		430	650	130	9.7			
replacement bulls	1	280	650	355		8.3		trivial
		165	650	890			7.0	
**total annual intake by one mother cow and her accompanying animals**								
kg dry matter feed					7500	7850	8100	
kg crude feed protein					1370	1060	860	
kg feed nitrogen					220	170	140	
**total beef production per one mother cow and her accompanying animals**								
kg edible beef y^{-1}					155	155	125	

[a] $\approx (0.9 \times 0.85 - 0.16)n_m$, slaughter at 600 kg or age of 2 y

[b] first pregnancy begins on first day in which age $\geq$ 310 d and weight $\geq$ 400 kg

kg of live weight yields 300 g of edible beef.

Per one mother cow, the herd thus yields 120–160 kg of total edible beef cow^{-1} y^{-1}.

G Estimating feed intake by the cattle herd

With diet composition specified (Table A), we can use NRC equations [15] to calculate the amounts of fodder consumed (better known as DMI or dry matter intake in the animal nutrition literature). While somewhat outdated, for the conditions envisioned here, the predictions of these authoritative equations are very similar to those of an updated version[21]. These expected amounts are given in table B.

H Estimating d, atmospheric nitrogen deposition rate

Early 21st century total atmospheric nitrogen deposition over the U.S. ranged [23] over 8-25 and 0.1-8 kg N ha^{-1} y^{-1} in the eastern and western halves of the country. Over 2014-2016[24], it ranged over 1-70 kg N ha^{-1} y^{-1}, with over half of all deposition rates falling inside 3.7-9.2 kg N ha^{-1} y^{-1}, distributed around the same mean and median rates, 6.7 kg N ha^{-1} y^{-1}.

These values are skewed toward the highly anthropogenically elevated eastern values. Much of this elevation is due to N volatilization in agricultural settings, whose elimination is a key environmental objective and a central goal of NSA. It thus makes no sense to consider those elevated deposition rates for the current purposes. Based on the more recent estimates of background rates not likely to be strongly biased upward by agricultural sources (Fig. 1a of

Walker et al.[24]), we expansively consider deposition rates in the 4 ± 2 kg N ha^{-1} y^{-1}.

I Estimating f_v, nitrogen fixing rates in the vegetal operation

Field peas fix [25, 26] 130-240 kg N ha^{-1} y^{-1}, and peanuts fix [27–29] 140-210 kg N ha^{-1} y^{-1}. Canadian pulses (dominated by dry field pea, lentil, and dry bean) fix [30] 50-150 kg N ha^{-1} y^{-1} with a mean of 95 kg N ha^{-1} y^{-1}, soybeans 60-200 (average 118) kg N ha^{-1} y^{-1}, and chickpeas [31] 60 and 20 kg N ha^{-1} y^{-1} under normal and drought conditions respectively.

Based on the above, under representative conditions f_v is expected to span roughly 40–200 kg N ha^{-1} y^{-1}.

J Estimating f_f, rates of nitrogen fixation in the fodder operation

Yang et al. [30] report nitrogen fixation rates of 27-141 kg N ha^{-1} y^{-1} in mixed legume and grass hay fields, and 141-300 kg N ha^{-1} y^{-1} in alfalfa fields, yielding respective means of 79 and 218 kg N ha^{-1} y^{-1}. Fixation rates in perennial temperate-to-high latitude alfalfa, red clover and white clover fields [36] were as high as 350 to 550 kg N ha^{-1} y^{-1}. These extremely high values, however, accompanied extremely high dry matter yields, as high as 12 metric tons ha^{-1} y^{-1}, with all y_f values scaling roughly linearly with dry matter yield when those yields exceed ≈ 2000 kg ha^{-1} y^{-1}. For the more modest dry matter yields—roughly in the 2-4 metric tons ha^{-1} y^{-1} range—that can be realistically expected for the low input,

extensive forage operations we envision, this data exhibited N fixation rates of 60-150 kg N ha^{-1} y^{-1}.

Comparing corn nitrogen uptake following wheat alone to the uptake following wheat mixed with clovers [37] reveals nitrogen gains rising extremely rapidly with unfertilized legume biomass near the end of the preceding season between 0 and roughly 1500 kg ha^{-1}, and much more slowly above 2000 kg ha^{-1}. Focusing again on the 2-4 metric tons ha^{-1} y^{-1} forage mass range, nitrogen gains are 50-65 kg N ha^{-1} y^{-1}.

Applying a similar indirect approach to 31 earlier analyses of corn and sorghum yields following fallow vs. leguminous cover crops collectively encompassing hundreds of individual measurements revealed [38] added post-leguminous productivity corresponding to the addition of 50-150 kg N ha^{-1} y^{-1}.

Multiple sources thus reveal a wide range of f_f values, with limited information about the distribution within the reported ranges. We thus forgo a standard measure of variability, and explore instead a range wide enough to bracket most observations, 50-250 kg N ha^{-1} y^{-1}.

K Estimating α, production rate of plant available manure nitrogen by a one cow "herd"

Numerous equations estimate the amounts of manure nitrogen a given diet (specified by composition and mass per day) is expected to yield. Angelidis et al.[32] report (their Eq. 1e presented in their table 2) the particularly skillful simple linear equation

$$\text{MNO} = -10.32 + 0.766\ \text{NI} + 0.108\ \text{TF}. \tag{S29}$$

Above, we use the original notation: MNO is an individual animal's manure (feces plus urine) nitrogen output in g N d^{-1}, NI is ration's nitrogen intake in g d^{-1}, and TF is the forage percentage in the diet, here 100. We estimate NI by dividing crude protein intake by 6.25, undoing the derivation by feed analysis laboratories of crude protein from the measured mineral nitrogen content of the analyzed feed based on assuming nitrogen constitutes 16% of bulk protein, and report it in the bottom numerical row of table B. Using these values and TF = 100 as inputs into Eq. S29 yields approximately 170, 130, and 100 kg of excreted manure nitrogen per one mother cow and her associated animals per year on the high, medium and low quality diet, respectively.

Because α denotes plant available nitrogen whereas the above values are excreted masses, they must be demoted for nitrogen losses due to volatilization from manure management facilities, imperfect manure collection, retrieval, and transpiration to fields, further post application volatilization N losses from fields, and failure of some applied manure nitrogen to be taken up by the growing crop. Taking the central value of reported estimates [33, 34], we set those combined losses to 60-70%, yielding generation rates of plant available recovered manure nitrogen by the one cow herd of

$$\alpha_{1,2,3} = 50\text{-}67,\ 39\text{-}52,\ 32\text{-}42 \left[\frac{\text{kg plant available manure N}}{\text{cow} \cdot \text{y}}\right] \tag{S30}$$

on the high, medium and low quality diet, respectively.

L Estimating β, cattle needs for forage nitrogen

From the bottom row of table B, a single mother cow and her associated animals require 220, 170, and 140 kg forage nitrogen annually on the envisioned high, medium, and low

quality diets. Inverting the exact values, this means

$$10^3\beta_{1,2,3} = 4.57,\ 5.89,\ 7.27 \left[\frac{\text{cow} \cdot \text{y}}{\text{kg forage N}}\right]. \tag{S31}$$

M Evaluating and independently checking $\alpha\beta$

With the above reported $\alpha_{1,2,3}$ and respective $\beta_{1,2,3}$, and because Eq. S29 is linear in NI, all three diets yield

$$\alpha\beta \approx 0.23\text{-}0.31 \left[\frac{\text{kg plant available manure N}}{\text{kg forage N}}\right]. \tag{S32}$$

This can be contrasted with analysis and modeling [35] of the full nitrogen balance and dynamics of four manure processing pathways in a Wisconsin dairy farm. Imperfectly for our purposes, these analyses address dairy, whose key element—high yielding, genetically hyper selected lactating cows—consume larger quantities of richer diets compared to their beef counterparts, and thus produce more and chemically and physically somewhat distinct manure. Yet the analyses address an intensive cattle confinement operation practically similar to the one we envision, with—most importantly—manure handling practices readily available for the considered beef operation. Consequently, and because their results are reported on a “per kg feed nitrogen” basis, these analyses are suitable if imperfect proxies for the cattle operation at the core of the envisioned combined farm.

With this caveat in mind, the analyses reveal mass ratios of plant available manure nitrogen output to feed nitrogen intake of 2.1, 2.2, 2.4 and 2.5 to 8.5 for the four considered manure management pathways, from which

$$\alpha\beta \approx 0.25\text{-}0.29 \text{ (kg plant available manure N) (kg forage N)}^{-1} \tag{S33}$$

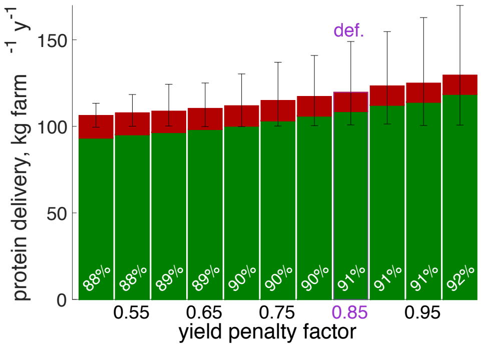

Figure F: Dependence of the model calculated protein availability on the assumed across-the-board yield penalty in the NSA system relative to today's conventional agriculture. The shown delivery statistics (mean and spread) are calculated over the three forage qualities and 250 Monte Carlo realizations. The spread whiskers span the 5th and 95th percentiles of the distributions, i.e., the core 90% of the distributions. Each bar comprises the plant contribution in green, with the beef contribution stacked over it in red. The fraction of plant protein in total protein delivery is shown numerically near the bars' bottoms, rising from left to right with declining vegetal yield penalty. The default yield penalty, 85% (the complement to the default 15% assumed yield penalty on which the main results are based), is highlighted in purple and with the "def." annotation.

follows.

Since this empirical $\alpha\beta$ range falls well within our calculated range (Eq. S32), we accept the α and β values given by Eqs. S30 and S31 respectively as suitably representative.

N Sensitivity of the results to the assumed yield penalty

Recall that in our model, there are two vegetal yield demotion factors. The first is the one handled by ξ (section B2). The second is the across-the-board 15% demotion of today's yields, to account for presumed lower efficiencies and yields in the envisioned NSA agricultural model relative to the mostly conventional production it strives to replace. This

assumed yield penalty is not based on any data, as the envision NSA system is novel and hypothetical, and its performance not covered by verified data. A reasonable guide is offered by organic agriculture, which is somewhat similar yet less stringent in terms of nutrient cycling. However imperfect the comparison may be, the assumed 15% yield penalty is slightly larger than observations of some specific organic systems [e.g., 39] and smaller than an estimated range for various organic operations [40]. Because of this uncertainty, in this section we evaluate the sensitivity of the NSA model results to the assumed yield penalty of the NSA system relative to the yields of current conventional agriculture. We do so by repeating the full calculations with assumed NSA yields of 50%–95% at 5% increments. This wide explored range easily exceeds the above organic estimates and most likely contain the unknown representative value for the NSA system. The metric we choose for evaluating this sensitivity is total protein a single ≈1.43 ha farm can deliver. The results are shown in Fig. F. Clearly, the assumed yield penalty impacts the solution very minimally, well within the expected fidelity of predictions made by such an idealized model.

References

[1] M. D. Tomer, T. B. Moorman, J. L. Kovar, K. J. Cole, D. J. Nichols, *Agricultural Water Management* **168**, 104 (2016).

[2] M. B. David, L. E. Drinkwater, G. F. McIsaac, *Journal of environmental quality* **39**, 1657 (2010).

[3] J. Blesh, L. E. Drinkwater, *Ecological Applications* **23**, 1017 (2013).

[4] J. A. Dittman, C. T. Driscoll, P. M. Groffman, T. J. Fahey, *Ecology* **88**, 1153 (2007).

[5] R. D. Yanai, *et al.*, *Env. Sci. & Tech.* **47**, 11440 (2013).

[6] N. A. S. S. United States Dept. of Agriculture, Quickstats (2019).

[7] E. R. S. United States Dept. of Agriculture, Feed grain yearbook (2019).

[8] G. Eshel, P. Stainier, A. Shepon, A. Swaminathan, *Scientific Reports* **9**, 10345 (2019).

[9] T. de Ponti, B. Rijk, M. K. van Ittersum, *Agricultural Systems* **108**, 1 (2012).

[10] V. Seufert, *Encyclopedia of Food Security and Sustainability*, P. Ferranti, E. M. Berry, J. R. Anderson, eds. (Elsevier, Oxford, 2019), pp. 196 – 208.

[11] A. R. S. United States Dept. of Agriculture, Fooddata central (2020).

[12] G. Eshel, *et al.*, *Nature Ecology & Evolution* **2**, 81–85 (2017).

[13] S. Kim, B. Dale, R. Jenkins, *Int. J. Life Cycle Assess.* **14**, 160–174 (2009).

[14] A. Mohammadi, *et al.*, *Journal of Cleaner Production* **54**, 89 (2013).

[15] N. R. Council, *Nutrient Requirements of Beef Cattle* (The National Academies Press, Washington, DC, 2000), 7th edn.

[16] N. Pelletier, R. Pirog, R. Rasmussen, *Agricultural Systems* **103**, 380 (2010).

[17] K. A. Beauchemin, H. H. Janzen, S. M. Little, T. A. McAllister, S. M. McGinn, *Agricultural Systems* **103**, 371 (2010).

[18] K. Beauchemin, H. Janzen, S. Little, T. McAllister, S. McGinn, *Animal Feed Science and Technology* **166-167**, 663 (2011). Special Issue: Greenhouse Gases in Animal Agriculture - Finding a Balance between Food and Emissions.

[19] M. J. Hersom, G. W. Horn, C. R. Krehbiel, W. A. Phillips, *Journal of Animal Sceince* **82**, 262 (2004).

[20] E. R. S. United States Dept. of Agriculture, Food availability (per capita) data system (2019).

[21] D. Fox, *et al.*, *Animal Feed Science and Technology* **112**, 29 (2004).

[22] A. Shepon, G. Eshel, E. Noor, R. Milo, *Environmental Research Letters* **11** (2016).

[23] L. Zhang, *et al.*, *Atmos. Chem. Phys.* **12**, 4539 (2012).

[24] J. T. Walker, *et al.*, *Science of The Total Environment* **691**, 1328 (2019).

[25] E. S. Jensen, *Plant and Soil* **101**, 29 (1987).

[26] G. R. . D. Corporation, Tips & tactics, nitrogen fixation in field pea, grdc grownotes southern, march 2018 (2018).

[27] M. J. Bell, G. C. Wright, *Australian Journal of Agricultural Research* **45**, 1455 (1994).

[28] S. Pimratch, *et al.*, *J. Agronomy & Crop Sci.* **194**, 15 (2008).

[29] T. J.Rose, L. J.Kearney, S. Morris, L. V. Zwieten, D. V.Erler, *Science of The Total Environment* **656**, 108 (2019).

[30] J. Y. Yang, *et al.*, *Agriculture, Ecosystems & Environment* **137**, 192 (2010).

[31] R. Abi-Ghanem, L. Carpenter-Boggs, J. L. Smith, G. J. Vandemark, *International Scholarly Research Network, Soil Science* **2012** (2012).

[32] A. Angelidis, *et al.*, *Agriculture, Ecosystems & Environment* **280**, 1 (2019).

[33] B. J. Zebarth, J. W. Paul, O. Schmidt, R. McDougall, *Can. J. Soil Sci.* **76**, 153 (1996).

[34] Z. Shi, *et al.*, *Field Crops Research* **127**, 241 (2012).

[35] H. A. Aguirre-Villegas, R. Larson, D. J. Reinemann, *Biofuels, Bioproducts, Biorefining* **8**, 770–793 (2014).

[36] G. Carlsson, K. Huss-Danell, *Plant and Soil* **253**, 353–372 (2003).

[37] S. Vrignon-Brenas, F. Celette, A. Piquet-Pissaloux, M.-H. Jeuffroy, C. David, *European Journal of Agronomy* **75**, 89 (2016).

[38] C. Tonitto, M. David, L. Drinkwater, *Agriculture, Ecosystems & Environment* **112**, 58 (2006).

[39] W. Cox, J. Cherney, M. Sorrells, *Agronomy* **9** (2019).

[40] V. Seufert, N. Ramankutty, *Science Advances* **3** (2017).
