# Supplementary material for: Small-scale integrated farming systems can abate continental-scale nutrient leakage
Source: PLoS Biol. 2021 Jun 3;19(6):e3001264. doi: 10.1371/journal.pbio.3001264 (PMC8174726; doi:10.1371/journal.pbio.3001264)
Supplement: Attachment — Submitted filename: response2reviews.docx [file pbio.3001264.s003.docx]

Gidon Eshel

43 Wynkoop Ln.

Rhinebeck

NY 12572

(413) 717-2187

[geshel@gmail.com](mailto:geshel@gmail.com)

Tuesday, September 22, 2020

Dr. Roland G. Roberts, Senior Editor

PLOS Biology

Dear Editor Roberts,

Thank you for handling the paper (PBIOLOGY-D-20-02394) and for your August 28 2020 decision email. Much appreciated.

As instructed in that email, here I respond to the *Nature Climate Change* reviews I shared with you. I reproduce the original reviews (complete with the typos in the original Review 1) below, and embed the responses in them, in easily distinguishable red.

Below, I use ms for manuscript, R1 and R2 to identify the two Reviewers, and RA to abbreviate “Regenerative Agriculture”.

Note that following the reviews reproduced below, we no longer use the term RA (which we used in the original *Nature Climate Change* submission). This enhances clarity and specificity, and reduces subjective ambiguity, for which we thank the Reviewers. Following comments by the *PLOS* *Biology* handling Editor and R1, we have also added to the Introduction a description of what the individual NSA farm looks like in practical terms.

Beyond these changes, in the course of responding to and addressing the Reviewers’ and Editor’s critique we have substantially revised the ms. We thank the Reviewers and *PLOS* Editor for their constructive, useful comments, which improved considerably the readability of this paper.

Thanks much again and all the best, Gidon

⎯⎯⎯⎯⎯⎯⎯⎯⎯⎯⎯⎯⎯⎯⎯⎯⎯⎯

**Reviewer #1 (Comments for the Author):**

Review of Eschel and Schlesinger for Nature Climate Change

Wwe are in the early stages of this regenerative agriculture movement, and sound science that accurately portrays regenerative food systems is desperately needed. From this paper, as written, it is not possible to assess its scientific rigor or the strength of its conclusions.

Moreover, although these conclusions are sorely needed to move forward, it seems premature to create these models as the scientific data used to support regenerative agriculture is largely lacking. I don’t disagree with the authors’ conclusions; practical experience in working in these regenerative farming systems shows that they produce a lot more food than conventional with reduced environmental impact. This paper simply does not offer the necessary support to make the claims that it does.

Reducing nutrient leaching from agricultural settings⎯and more broadly reducing the environmental burdens food production exerts⎯is indeed needed, and is part of the reason we wrote this paper. We disagree with R1’s belief that “it is not possible to assess [the paper’s] scientific rigor or […] strength of its conclusions.” The paper is based on a novel model, which is derived fully and with complete transparent details in the Supplementary Information. Could it be that R1 did not see, or was not given the SI file? While one may challenge specific aspects of the derivation of the governing equations⎯and there are aspects of the paper that while cogent and logical are imperfectly constrained by observations⎯assessing the paper’s scientific rigor and the strength of its conclusion is readily permitted by the extensive, detailed derivation of all aspects of the governing equations. To account for the tendency of some readers and Reviewers to ignore the Supplementary material, and to take advantage of the *PLOS Biology* flexible publication guidelines, we have augmented the main text with considerable additional details of the calculations so as to endow the paper’s main text with a bit more independence. In most cases, the added text is not in lieu of Supplementary material, but in addition, with minimal redundancy.

Re “…the scientific data used to support regenerative agriculture is largely lacking”: we neither “support” nor oppose regenerative agriculture or any of its competitors and alternatives. The purpose of this paper is not to take sides in a political or even a scientific polemic. We simply quantify the nutritional and environmental impacts of one implementation of a nitrogen-sparing farm model. If in the above sentence R1 stresses that it is not clear whether RA is superior to alternative models, we potentially concur but remain agnostic until corroborated or refuted by quantitative analyses such as ours.

A major problem with the paper is that the treatments are never defined.

There are no “treatments” in this paper. It is not based on empirically comparing performance of agricultural systems under distinct treatments, as typically used for comparing organic to conventional, say. This is a model-based paper, and the model *is* clearly defined and derived in full details. Its results are then compared simply to today’s US agriculture characterized by USDA data. The above details are now far more clearly stated in the revised paper, with more explicit references.

Regenerative agriculture is not simply mono-cultures of soy, corn and beef that eliminate agrichemical use. Indeed, it is impossible to understand how regenerative systems are defined in this paper at all. It looks like the authors define it as conventional corn and soybean monocultures that replace chemical fertilizers with manure generated from a hay-based feedlot.

We do not discuss corn or soy monocultures; “corn”, “soy”, or “mono” do not appear in the ms. R1 argues against points we do not make. Moreover, the description of the Monte Carlo procedure makes clear to the reader that crop diversity, the antithesis of the monocultures R1 imagines and protests, is key to the success of the envisioned system and is strictly enforced.

This is NOT a regenerative system. A regenerative system increases soil health and increases biodiversity.
Since RA is not used in the current ms, the first point is no longer relevant. While soil “health” is ill-defined, RA is indeed *designed* to enhance soil organic carbon. Yet as R1 points out, RA is currently not sufficiently well-observed to definitively ascertain either assertion. They thus await corroboration or refutation by future observations.

Confined animal operations are largely eliminated from regenerative systems. Monocultures of corn and soybeans are also largely eliminated from these systems. The manure that fertilizes the “regenerative” cropland is generated in feedlots? As stated in the paper, yes. And then is the sole source of fertilizer for the monoculture cropland? Does this account for the reduced requirements of regenerative cropland for fertilizers? To my knowledge, all regenerative
operations increase their soil health and need much less fertility inputs. Again , soil “health” is ill-defined. If⎯as R1 appears to suggest⎯after some years under the modelled practice fertilization needs will drop, which is at this point an anecdotal possibility, then productivity and outputs will rise. If this possibility comes to pass, therefore, it would strengthen the presented results. At this point, however, this is too speculative to assert, which is why we refrain from doing so. Similarly, what is a “regenerative diet”? Eating more corn and soybeans? Humans consume very little of these crops- most are fed into cattle or into our cars as biofuels. Without a much clearer definition of the treatments, this paper’s conclusions cannot be evaluated. For example, where did the 60-80% increase in protein provided by regenerative systems come from? I am assuming that it is coming from the “vegetal” component of the regenerative system? The authors do not seem to be thinking about the regenerative treatment as a totally new system.

The resultant diet is defined as follows (taken verbatim from the text, around line 127): “The NSA diet is the resultant overall daily per capita food availability, the sum total of beef plus plant output of the NSA model deployed over the 105 million ha of U.S. cropland enjoying ample precipitation (excluding croplands in the USDA agricultural districts of Southern Plains, Mountains, and the entire Pacific seaboard), divided by 330 million Americans times 365 days a year.”

Relatedly, the questions R1 raises indicate that the writing could have been clearer. In that, R1 agrees with the *PLOS Biology* Editor. We agree; a clearer description of both the individual “atom” farm and of the full continental-scale deployment of variants of this farm in the full envisioned system would indeed be helpful. Our earlier treatment of this was insufficient. We thus augmented this discussion. We moved the schematic of the basic farm unit to now being Fig. 1, and added considerable text (as opposed to earlier, when this was only in the SI) describing the individual farm and its governing principles. This now occupies the final few paragraphs in the Introduction.

The next critical problem is that it is unclear where the data for the paper comes from. Where did the data come from to drive these models? As stated in the main text and described in details in the SI, this is a mathematical model. Since it is not an ODE-based model, it has no boundary conditions, and therefore is not “driven” per se. At the same time, in an indirect way the question still has some relevance, if instead posed as “how are model parameters quantified? What guides their values?” These are clear questions, whose answers are similarly clear: every parameter has a corresponding section in the SI, where the detailed logic guiding its derivation is introduced. There are no citations for where the information comes from. Every assumption is clearly referenced in the relevant SI sections. It is not possible to assess the strength of the model without it being nested in reality. As I mention above, there is little data from truly regenerative operations available, and one cannot simply take data from conventional operations that feed hay instead of grain to their feedlot. The same numbers do not apply to a regenerative farm. Just as one example: where did the data for the nutrient analysis in Figure 2e come from? What are these nutrient differences based on? Plant-based diets? Hay-based cattle production? The caption of Fig. 3 clearly refers the reader to Supplementary section S18, where these questions are clearly answered.

I appreciate that the authors are trying to support the regenerative agriculture movement, and that they are willing to tackle a very challenging problem of trying to predict the full impact of regenerative agriculture on society and the environment. But I don’t think that this paper accomplishes their mission of creating a rigorous comparison of clearly defined systems. We do not support or challenge any particular approach or model.


**Reviewer #2 (Comments for the Author):**

We thank R2 for his careful reading of the paper’s various parts and for his thoughtful, illuminating comments. Below we describe how we modified the paper in accordance with R2’s comments, for which we are very thankful.

This paper develops a theoretical model for a tightly coupled system of mixed crop-livestock production in the United States, under the rubric of regenerative agriculture. The study quantifies the amount of food that can produced using their model of agriculture as well as some of its implications. The authors show that, using less than half of today’s agricultural land, their model agriculture can produce 60-80% of beef and 135-140% of protein, while averting most synthetic fertilizer use and some greenhouse gas emissions. The implied diet is, however, more plant-based than today’s diet. The paper has a lot of interesting features and novel contributions. However, I have several concerns that I feel should be addressed before it can be considered for publication. We like the above synopsis, thank R2 for these positive comments, and address the concerns one at a time below and in the rewritten paper.

• First, I feel that your use of the term ‘regenerative agriculture’ is misleading. When I agreed to review the paper, I thought it would be about carbon cycling, not nitrogen cycling. Nowhere in the title or abstract is it clear that the core of this paper is about nutrient cycling. The whole argument about regenerative agriculture, especially as it relates to beef production, is about carbon sequestration in the soil, with some arguing that it can even be carbon neutral or negative by sequestering more carbon in the soil than carbon and methane emitted from energy use or enteric fermentation respectively. Therefore the title, abstract, and framing of this paper, as being about regenerative agriculture is a little misleading. An alternate framing, about mixed crop-livestock production, is more appropriate in my opinion (see, for example, Herrero, M., et al., 2010). I think such a reframing will not only make the purpose and underlying work of the paper more clear, but it will also help address my next main concern.

True and well put. We concur. Therefore (1) we eliminated the term Regenerative Agriculture form the paper, replacing it with “nitrogen-sparing agriculture” or NSA for short. And (2), we now explicitly use the term “mixed use farm”, which is indeed a good short catchphrase for the system we describe, and cite the Herrero paper, which *is* an excellent fit yet was missing from the earlier version. We do believe both changes discernibly improve the paper, for which we thank R2.

• Another related comment to the above is that other papers (e.g., Mueller et al. 2012) have considered a reconfiguration of current conventional systems to be more efficient in terms of water and nutrient use efficiency. Others (e.g., Springmann et al. 2018) have optimized both production practices and diets to achieve greater efficiencies. Both of those papers are global (not US specific), but neither invoked regenerative agriculture, and argued for reconfiguring conventional production systems. How does your work compare to these studies?

We agree, indeed failing to cite this paper⎯even though Nathan and I (GE) have just recently given back-to-back talks at Friday Harbor Lab⎯was an omission on our part. We now cite two of Nathan’s papers that are indeed pertinent to our effort here.

Re Springmann et al. (2018), first, clearly that too is a key reference whose omission earlier was an oversight on our part, now corrected. We briefly compare the two results in newly added discussion a bit after Fig. 2.

• The core question being asked by the paper, on whether regenerative agriculture can be scaled up, is not the most important question in my mind. The most important question about beef production through regenerative agriculture today is about the carbon neutral argument mentioned above. So while the authors argue that the question of being ‘environmentally sound’ is moot if it cannot be scaled up, I would counter-argue that the question of scaling up is moot until it can be proved to be environmentally sound. Given this context, once again, I think reframing the paper to be about tightly coupled nitrogen cycling in mixed crop-livestock systems makes more sense. That argument has been made for decades, including most eloquently by Wendell Berry (1996).

First, as already stated in our response to R1, following this point and similar ones made earlier by R1 we have now more clearly (and less misleadingly) call the envisioned system “nitrogen sparing agriculture” (NSA), no longer “regenerative agriculture” (RA). Re which of the two questions is more important, we certainly wholeheartedly agree with R2 that the question he poses is *also* important. Both questions are key, both merit close scrutiny, and both must be quantitatively answered before we can all get behind NSA, RA, or any of their many similarly motivated alternatives. Since one can easily *assume* certain answers to one of the two questions and then critically examine the other, there is really no methodological impediment to analyzing each individually. And examining NSA’s scalability and nutritional desirability *assuming* economic viability is what we do here. In the revised paper, we address this duality briefly in a newly added discussion (centered around line 90) shortly after Fig. 1.

• The counterfactual calculations are still unclear to me after reading the paper and the supplement a couple of times (although I must admit that I did not manage to read the supplement in great detail, beyond understanding the governing equations, and some of the model boundary conditions). In figures 2 and 3, where do the data shown in dotted lines, representing the baseline to which the model is being compared, come from? R2 is correct, the origin of these were not properly stated and cited in the earlier version he reviewed. This is now fixed, thank you. There seem to be two different sets of baselines, calculated in different ways, if I understand correctly. The nutritional ones (2a,2b,2e,3) seem to based on currently reported data (i.e., current US data), … Exactly, now clarified and expounded on in a revised nd augmented caption to Fig. 2 …while the environmental ones (2c,2d) seem to be based on a counterfactual calculation of fertilizer use and GHG emissions that would have occurred had model diet been produced using conventional methods (lines 217-218 of supplement). If I understand this correctly, I need to ask whether these comparisons are consistent. The model world is different from
the real world in at least two major ways: 1) The way the food is produced has changed; and 2) The diet itself has changed. (There are other differences I think, which is discussed in the next point.) The baseline for the nutritional calculations accounts for both of these, while the environmental baselines only account for the 1st shift.

First, R2 got the meaning of the nutritional reference dotted lines exactly aright. The meaning of the environmental references (dotted horizontal lines in Fig. 2c,d) is now clearly stated in the revised and augmented caption of Fig. 2, with the relevant refs. The way the caption was worded earlier was confusing and unintentionally potentially misleading, as R2’s confusion indicates. We hope and trust the new, shorter and more accurate caption resolves this issue. A different sloppy wording also prevented the caption of the earlier Fig. 3 from simply and succinctly explaining what the dotted line there means. Thankfully, it is in fact very simple, the dotted lines in Figs. 2b and 3 are one and the same. The offending caption is now reworded, thank you.

R2 is correctly describing the calculated values (bar heights) in Fig. 3 (as distinct from the national reference, the dotted horizontal line), but reveal important confusion. It stems from our use of the word “reference” for the red bar, understandably confusing R2. We used “reference” to mean “the main protein delivery, reported in Fig. 2b, to which we compare the protein delivery of alternative allocations considered in Fig. 3”. But R2 understandably confused this use of reference (the red bar) with the horizontal dotted line in Fig. 2b, which is *also* provided as a reference value. We now rewrote the caption of Fig. 3 to alleviate this needless confusion our earlier wording caused.

With this correction, the answer is: yes, R2 is correct about the two changes occurring at once in the model; and yes, the comparisons are internally consistent. In Fig. 2 the horizontal dotted lines are the national means that characterize today’s US food system, whereas in Fig. 3 100% is not what we have today, but the basic result of our calculation, the average of the three bars in Fig. 2b. This should now be clear, and thank you for bringing this issue to our attention.

• What about trade? A large portion of current US crop production is exported, and is not meant for US domestic consumption? This paper assumes that all production from this model is devoted to domestic consumption. So I am concerned that the baselines used for the nutritional calculations are not the right ones because current availability in the data is after accounting for exports and imports. Also, what about food waste? Do those baselines include food waste, while your model world has no food waste?

Yes, R2 is exactly right, disallowing for trade *is* a limitation of the presented calculations. We now make that clear and discuss it briefly in a newly added paragraph shortly before the end of the Introduction (around line 140).

• I have a broader question about your use of the term “scaling up” in the paper, which I think you are using in a narrower sense than generally used. What you have done in the paper is defined a 1.43 ha farm, with tightly linked nitrogen cycling, and calculated what it can produce per ha. Then you have multiplied that by 105 million ha. But when people normally think about scaling up (e.g., scaling up organic production), they are typically talking about a suite of different considerations including economic (costs of production, implications for food price, and implications for demand), infrastructural (supply chain configuration, setting up markets, etc.), policy (reforming the Farm Bill so that incentives are shifted, subsidies removed, etc.) and so on. Those are the real challenges of scaling up, which you have not even touched upon in the paper. How do you propose really scaling up your model of agriculture in the real world context?

This is a good point, indeed scaling up is far more societally involved than simply creating national polymer out of this 1.43 ha monomer, which is what we do here. Agreed. But these processes are not our focus, we are not economists nor policy experts. We therefore briefly address this issue now in the expanded Introduction, and call our results “upper bounds” in the sense that reality will likely be somewhat less productive than what we have, but leave it at that. Hopefully, an agricultural economics group will take up this issue in a follow-up to this paper.

• A key assumption in the model is the 80-90% nutrient efficiency (compared to 50% for conventional). A lot more justification is needed for this. If you relax this parameter, I suspect your model results will completely change. In other words, by specifying this parameter, it feels like you have essentially “put the answer in” to the model in relation to nitrogen cycling. Granted, the most interesting outputs are related to the food production from the model.

While R2 is making a very reasonable and understandable assumption here, it is not so. To demonstrate this rigorously, we have added a large sensitivity calculation, whose results we now present and discuss in a newly added Supplementary sub-section S1.1 and in a new Figure S1 therein. The broad conclusion is that most results are very minimally sensitive to nitrogen cycling efficiency in either the vegetal or forage plots, but that beef output⎯while also modestly dependent on cycling efficiency in the forage plot⎯is more sensitive, primarily because it is a fairly small output under any reasonable assumed cycling efficiency, which makes relatively small changes amount to larger *relative*  changes. In any case, all sensitivities are acceptable given the idealized nature of the derived model.

Minor comments
• You have assumed a yield penalty of 15%, referencing literature comparing organic and conventional yields. The estimates range from 19 to 25% (see Seufert & Ramankutty, 2017).

This is a very good point, a point important enough to merit a newly added Supplementary section, S19 (to which we refer the reader in the Results section of the main text). Please see that new section, we believe it addresses the issue in a manner that will satisfy R2. We thank R2 for raising the issue as we had the calculation presented in this added section early on, but chose to exclude it from the paper for fear of overwhelming readers. On closer examination propelled by the above point, we changed our mind. Thank you for that.

• Why do you compare averted GHG emissions to the national total, and not just the agricultural component? This was not entirely clear to me.

Yes, that is not obvious. We do so to give a *general* sense of the order of magnitude import of the calculated averted emissions. As R2 knows well, at least when disregarding the IPCC LULUC emission category, agriculture directly accounts for a very modest fraction of all national emissions. Consequently, simply percent reduction in *agricultural* emissions is poised to deliver a grossly inflated view of the societal betterment the NSA system is poised to deliver. To avoid such misleading conclusions by casual readers less attuned to the above details, we favor the national total.
• I should also add that the paper was more challenging to read than I imagined. Part of it has to do with the clarity of writing. For example, take lines 198-200, I still am having a hard time parsing that sentence, its connection to the previous sentence, and relationship to that entire section (although I do understand figure 3 and the corresponding analysis).

We are sympathetic to this comment; the earlier, reviewed version was indeed at times dense. We hope and trust R2 will find this extensively rewritten version, in which we made a very concerted effort to enhance readability, a lot easier to follow. Thank you.

References
• Berry, The Unsettling of America : Culture & Agriculture, 1996, p. 62: “Once plants and animals were raised together on the same farm — which therefore neither produced unmanageable surpluses of manure, to be wasted and to pollute the water supply, nor depended on such quantities of commercial fertilizer. The genius of America farm experts is very well demonstrated here: they can take a solution and divide it neatly into two problems.”
• Herrero, M., et al., Smart Investments in Sustainable Food Production: Revisiting Mixed Crop-Livestock Systems, Science, 327(5967), 822-825, 10.1126/science.1183725, 2010.
• Mueller, N. D. et al., Closing yield gaps through nutrient and water management, Nature, 490, 254-257, 10.1038/nature11420, 2012.
• Seufert, V., and N. Ramankutty, Many shades of gray—The context-dependent performance of organic agriculture, Science Advances, 3(3), 10.1126/sciadv.1602638, 2017.
• Springmann, M. et al., Options for keeping the food system within environmental limits, Nature, 562(7728), 519-525, 10.1038/s41586-018-0594-0, 2018.


Navin Ramankutty
