# Supplementary material for: Small-scale integrated farming systems can abate continental-scale nutrient leakage
Source: PLoS Biol. 2021 Jun 3;19(6):e3001264. doi: 10.1371/journal.pbio.3001264 (PMC8174726; doi:10.1371/journal.pbio.3001264)
Supplement: Attachment — Submitted filename: response2reviewsV2.docx [file pbio.3001264.s005.docx]

Gidon Eshel

43 Wynkoop Ln.

Rhinebeck

NY 12572

(413) 717-2187

[geshel@gmail.com](mailto:geshel@gmail.com)

Thursday, February 25, 2021

Dr. Roland G. Roberts, Senior Editor

*PLOS Biology*

Dear Editor Roberts,

Thank you for handling the paper (PBIOLOGY-D-20-02894R1) and for your December 14 2020 decision email. Much appreciated.

As instructed in your email, here I respond to the reviews you shared with me. I reproduce the original reviews below, and embed my responses in them, in easily distinguishable red.

Below, I use ms for manuscript, and R*n* to identify Reviewer *n*

I thank the Reviewers and PLOS Biology Editors for their constructive, useful comments, which improved considerably the quality and presentation of this paper.

Thanks much again and all the best, Gidon

⎯⎯⎯⎯⎯⎯⎯⎯⎯⎯⎯⎯⎯⎯⎯⎯⎯⎯

**Preliminary general author response**

These reviews have been among the most useful, thoughtful I ever got. I really appreciate the productive expert input!

The flip side of this statement is that the probing critique made me completely rethink the structure of the model. Indeed, the resubmitted ms is based on an almost entirely distinct model formulation, one that most often closely tracks the reviews. While the results are qualitatively unchanged, and quantitatively quite similar to those of the initial submission (which is very reassuring), the new model is far simpler to introduce and derive. This means that the completely revised SI file has dropped to $\approx$half its original length, an added bonus. The main text too is extensively revised, all figures are redrawn to reflect the new results, and some are redesigned.

Following the uncoordinated yet universal agreement among the Reviewers that the assigned nitrogen retention rates of the previous version (in which fixed 80% and 90% of the available nitrogen in the vegetal and forage subunits transformed into respective yields) are unpersuasive, in the current formalism the nitrogen retention rates are state variables, dynamically valuated within the solution procedure. I too find this far more elegant, and I am thus particularly thankful for this input.

Another key difference from the earlier model is the treatment of *γ*, the manure partitioning coefficient. In the early version, I derived a complex formulation for dynamical valuation of *γ* as a key state variable. The reviews, plus the observation that in most cases this dynamically valuated *γ* stubbornly lingered around 0.98-1.0, which I made during the revision process, made me rethink this. This inquiry has now culminated in *γ* =1 throughout except in sensitivity analyses. This assignment is based on a simple analysis of optimality, now described in the SI. This change too made for far simpler verbal presentation and code (which will become part of this paper’s SI).

A final important difference between the resubmitted and the initially submitted work is the treatment of yields’ dependence on available nitrogen input, the need for which was highlighted in Prof. Billen’s (R1) review. See below, and the new SI, for details.

Beyond this general preface, my responses follow, with many thanks to the Reviewers and Editors for their incredibly useful input.

Reviewer #1:
[identifies himself as Gilles Billen]

 I liked very much this paper, as well as the previous ones by the same author: their provocative prospective views of what alternative food systems could look like are very interesting and stimulating.  The concept of « nutrient sparing agriculture » (NSA) with the objective of zero industrial fertilizer use and full closure of nutrient cycles is quite useful. 

The demonstration that the generalization of this model offers a realistic alternative to today's industrial agriculture is quite convincing.

I am impressed by the degree of details in the link the author is able to establish between his hypothetic agricultural system and the diet, described at the level of micronutrients. 

Thank you so much, Professor Billen, I am honored!

I have however some objections to the formulation of the model, from an agronomical point of view.

(1) The author seems to give no consideration to crop rotations. In the "v" part of the farm, the different crops are distributed randomly, and independently of each other. Their Nyields are defined a priori, as constant values, independently on the other, and on the applied fertilization.  Similarly, in the separated "f" part of the farm, symbiotic fixation is constant for a given forage quality.  In the real world, fodder crops and the different "v" crops alternate on the same land, and that is how nitrogen fixing plant can sustain the growth of the succeeding crop (in addition to manure transfers).   Here, if I understood correctly,  yv is determined by the basket of vegetal production and this determines the amount of manure required.

That is an excellent and fair point; because agricultural systems are ecosystems, their various occupants interact and impact one another’s productivity. Surely. Beyond strong agreement, I have several responses to this.

First, note that following this comment, I reformulated the treatment of the vegetal yield in the model. The discussion of this is now first introduced near the opening of the rewritten section S2, and is further bolstered in more detail in (the also rewritten) section S2.2.

Second, the solution strategy, Monte Carlo randomization, is explicitly designed to heed this truth and take note of it. The key corollary of the potential of crop rotations to impact productivity is a wide distribution of farm productivities, with relatively few exceptionally high or low, and most near average farms in between the two extremes.

Because particularly productive or counterproductive crop combinations/successions raise or reduce mean yields, the above farm distribution will result in a correspondingly wide spread of productivities. *Agronomy* is laser-focused on identifying and promoting the successful practices and demoting failing ones. But here, the focus is environmental, not agronomic. The question therefore becomes: Is the above spread of yields adequately represented in the mean and spread of the presented result? The answer is yes, all are well represented by the suite of particular and mutually distinct monte Carlo realizations. Now Prof. Billen may well counter this, reasonably, by arguing that in the model, this is represented by a randomization fiat⎯the number (mostly inside [0.45,0.98]) by which the basic crop-specific yield is multiplied in a particular Monte Carlo realization⎯while in reality this happens for specific combinations of crops in particular order. True again. But identifying winning rotation practices⎯the crux of the matter in *agronomy*⎯is a side issue environmentally. Does the randomization therefore faithfully represent the *statistical properties* of the environmental impacts of this? I believe, and I am quite sure Prof. Billen would agree, that it *absolutely* does in that the statistics the calculation ultimately generates incorporate the effects of both particularly skillful and clever implementations of crop rotations as well as, alas, the inevitable and equally rare especially *un*successful ones. And that faithful statistical representation is what matters here, leaving the agronomic question to, well, agronomists, an honor I do not claim...

Finally, my numerical formulation of the idealized modelled “farm” should not be associated with fixed, unchanging real estate, but only with an unchanging area, wherever in the farm it may occur. I now try to make this clearer in the paper with the following addition (around line 100 in the revised ms). “While their time-average areal extents (“acreage”) are unchanged⎯1 and $\approx$0.43 ha of vegetal, and forage production respectively (Eq. S16 and section S2)⎯the geographical boundaries of the subunits are not rigid, so as to optimize nutrient dynamics. This may mean, e.g., allowing temporary post-harvest cattle grazing on either of the two crop-based subunits, exploiting such marginal resource as minor hay regrowth or aboveground residues, while replenishing nutrient stocks via direct manure deposition. It can also mean deliberate alternating between vegetal and fodder crops on a given plot of land so as to optimize the role of nitrogen fixers, a task made challenging by distinct required bed preparations compounded by no- or low-till dictates [47].”

(2) N inputs through symbiotic fixation are all but natural inputs:  these are the levers for intensification of the system, and not a natural constraint as considered in this model.

This too is a fair point. In response, I rewrote each of the offending sentences. I replaced “natural sources” with, more explicitly, “symbiotic fixation and atmospheric deposition” and “the modelled farm receives only natural atmospheric sources of…” with “the modelled farm receives only atmospheric sources of…”. Finally, I reworded the caption of Fig. 3 with alternative wording consistent with this point.

(3) The model assumes linearity of total Nyield (vf and yv) versus total soil Ninputs (equ S1 and S2), and discusses the proportionality (or leakage) coefficient (0.8 - 09) as constant N use efficiency.  Yet in the real world, the yield fertilization relationship is not linear, and tends to plateau at higher rate of N inputs.  This is the cause of increased environmental losses for intensive systems: the leakiness of a cropping system is a function of its level of N inputs. The assumption of linearity of the equation prevents a correct estimate of the environmental losses which are at the core of the paper.

There is clearly no doubt about the validity of this point. Because of this, I replaced this strong fixed assignment with a nonlinear “one minus Michaelis–Menten”-type parameterization of the leakiness parameters. I turn Prof. Billen’s attention especially to the new section S2.2, Eq. S24 therein, and the discussion surrounding it. I also now include an explicit sensitivity analysis (section S14) to both the structure of the parameterization and, independently, for the parameter values chosen. The results show clear robustness under this admittedly imperfect formulation.

(4) This is more a sentimental objection: I hate the idea of confined livestock farming! Outdoors grazing is, and I hope will remain, an essential part of our traditional landscapes….

Of course, of course, agreed. But here, I am trying to keep my scientist hat firmly on, and check my long-distance bicyclist sentiments, which Professor Billen so nicely mirrors in this comment, at the door in the name of impartiality. Let’s calculate first, and form an opinion subsequently.

Reviewer #2:

This paper presents the results of a modeling exercise that calculates the optimal farm size for minimizing nitrogen use (N sparing agriculture) then scaled to enough arable cropland in the US to meat American dietary needs. The model farm is highly simplified to 3 components: forage production, food production, and animal production. Nitrogen is spared by N fixation sufficient to replace N exported in crop and beef harvests plus within-farm recycling of that used for animals via manure. Crops are random mixtures of those grown in the US chosen to meet human nutritional requirements. Environmental N losses are based on nitrogen use efficiencies of 80% for food crops and 90% for forage. The results of monte carlo simulations support many other analyses showing that reactive N losses can be minimized by moving populations to a plant-based diet. What is unique is its also doing so by reducing beef production by only 20-40% rather than 100%. However the highly simplified nature
of the analysis begs the significance of this finding and there needs to be a more robust analysis of the factors that allow this inclusion.

This is, in fact, is a hard paper to read: it's not well structured and there is a lot of opinion and loose rationalizing and general wordiness in the narrative, overuse of references, insufficient information on nitrogen fluxes, and missing figure legends. In other words, it needs a good editing.

Re the writing, I do see the point, fair enough. I therefore completely rewrote and restructured the paper. I tried very hard to find uncorroborated opinions and weed them out. I also toned down in several points in the paper potentially controversial statements I thought R2 *may* have been addressing in this statement. Most notably, this spirit impacted and guided my thorough rewriting of the Introduction.

Re references: on fresh reading, I agree, references in the earlier version too often interrupted the flow. While I eliminated a handful but kept most references, in recognition of the important contributions on which my work builds, in response to the above comment I rearranged many of the citations so that they are now combined into *ranges* permitted by PLOS Biology citation style yet markedly less obtrusive than they admittedly sadly were before.

That said, to me the chief scientific weakness appears related to N use efficiencies, central to the paper's overall conclusions and unrealistically high for even organic systems like these. Choosing average values between 50% (typical for fertilized field crops) and 100% (based on Hubbard Brook, an aggrading forest) isn't sufficient without further quantitative justification. Values should be based on average (not cherry picked) literature values, area-weighted for the different types of cropping systems. Moreover, most of the food crops in these systems appear to be horticultural - with NUEs often well below 50% regardless of N source.

This point is very well-taken, and almost identical to an earlier one by Prof. Billen. Since I already responded, please see my response above. R2 and Prof. Billen are both right. The short of it is that this is completely rethought, recoded, reformulated in the current resubmitted version of the ms. I hope Prof. Billen and R2 will find the new treatment of this important parameter satisfying.

I should add that if not already, the paper should be reviewed by a modeler who can fully evaluate the derviced equations in supplemental material.

Specific comments
1.      throughout, the word "vegetal" is an odd usage to denote food crops, i.e. used for direct human consumption. I've been reading the agricultural literature for decades and this is the first time I've encountered it.

I am happy to consider proposed alternative(s). I don’t know of any, except “plant-based items”, say, which is a mouthful. Meanwhile, I checked [the Merriam-Webster dictionary](https://www.merriam-webster.com/dictionary/vegetal) and was affirmed that, yes, this is indeed a legitimate word, whose meaning is precisely the one I was going for. I would like to keep this succinct word in place. On the other hand, if the Editors are in agreement that this word is awkward, I am happy to entertain proposed alternatives.

2.      the Abstract is 50% introduction. Better to spend the space describing the model and its major assumptions.

R2 is right here. In response, I shortened markedly the Abstract’s first half or so that was indeed more introductory. It is now 251 words, compared with just under 300 in the initially submitted paper. Progress! At the same time, I wrote the paper after careful examination of numerous recent *PLOS Biology* papers. In response to this comment, I re-examined the issue. I point R2’s attention to the fact that in that cohort of recent *PLOS Biology* papers, many abstracts are in fact partly introductory (call it the *Nature* and *Science* effect?!) Take, e.g., Nagelkerken et al. [*Ocean acidification boosts reproduction in fish via indirect effects*](https://journals.plos.org/plosbiology/article?id=10.1371/journal.pbio.3001033), January 19, 2021; roughly the first third of the abstract is an intro to the issue at hand. Same goes for Ginsberg et al. [*Why Lyme disease is common in the northern US, but rare in the south: The roles of host choice, host-seeking behavior, and tick density*](https://journals.plos.org/plosbiology/article?id=10.1371/journal.pbio.3001066), January 28, 2021. The current, shortened abstract thus seems to fit right in. Beyond this, I am happy to defer to the Editors for a final determination.

3.      line 71. Figure 1 seems almost unnecessary in its simplicity. The image of CONUS and center pivot irrigation circles are confusing - the modeling is not geospatial and in any case not irrigated; better to simply represent the 3 farm elements with some explanatory arrows denoting major nitrogen flows.

Agreed. Changed accordingly. Thank you.

4.      line 65 - what does sequestration limiting mean. Nitrogen per se has little direct impact on carbon sequestration if that's what is meant.

This sentence is no longer in the paper. But the issue that sentence strove to address is still discussed in the rewritten and reorganized Introduction, albeit differently, bolstered by pertinent references. Since I am not a biogeochemist, I ran this argument by Bill Schlesinger, who basically approved but had several comments. All are now addressed in the new version, and I am quite confident R2 will find it satisfactory now.

5.      line 115. What exactly is 1.43 ha - the entire individual farm unit?

Yes, exactly. It is now made clearer and more explicit than earlier, both in the main text and⎯in more detail⎯the SI.

6.      line 261. The modeling is central to the paper and deserves describing in Methods, not buried in the supplemental material.

I agree. Yet judging by several recent papers, whose Methods sections are rather brief and non-technical, and in which the technical crux is therefore relegated to the SI, this does not seem to be *PLOS Biology* style. If the Editors authorize a technical, equation-laden Methods section, I will gladly move parts of the SI to the Methods section.

Reviewer #3:

The paper claims that substantial environmental benefits (reduction in nutrient loss, greenhouse gas emissions) could arise from a shift towards NSA (Nitrogen Sparing Agriculture) in the United States. While these claims are not novel (the author cites several papers that make the case), as far as I am aware the national perspective, for the USA, is novel.

I agree with R3 that this indeed novel for the U.S. But I think this paper is more than that. Many of the papers advocating various models of livestock-centric “sustainable agriculture”^[1]^ tend to be heavier on “activist” rhetoric than on technical rigor or quantitative numerical reproducibility. Some are outright misleading, a point richly established in the [literature](https://www.ars.usda.gov/ARSUserFiles/4472/RANGELANDS-D-13-00044.pdf)^[2]^ and summarized in Nordborg’s 2016 [*Holistic management–a critical review of Allan Savory’s grazing method*](https://orgprints.org/34330/1/holisticmanagement_review.pdf)^[3]^. Ameliorating this sorry state is my overarching motivation here. The development of a fully documented numerical model with which to examine this general suite of questions, an examination that can go on for as long as it takes because its full technical details and governing equations are fully documented in the SI of the current paper, and the code on which it is based is made publicly available, is a non-trivial step forward. The simple model I report here is definitely not the final word on this research thrust, but that is the point: the more technically rigorous follow-ups on this paper, the more it will evolve and improve. And the more the community relies on such scientific methods⎯with conclusions that can be readily falsified or corroborated by future work⎯the less qualitative our discourse will be and the more reproducible its key tenets will be.

I am very sympathetic to the general idea - from an economic perspective it is my view that we - I am writing in Europe - are too far to the right on our agricultural response curves, particularly for manufactured nitrogen. I cannot comment on the USA, except to note that I assume that you continue to be 'land rich' and thus intensity per acre will be lower for fertilisers than in Europe. However, the argument probably still holds. The paper would be of interest to academics and policy makers as it takes farm level effects of attempting to close nutrient systems - essentially, specialise in (intensive) beef production and
use manure as fertiliser for a range of field and tree-based vegetables and fruits - to the national level. If this less leaky system 'works' at the farm level, why not extend it? This is also the main flaw in the paper - if the system results in a vector of environmental effects on one acre, the effect of many acres is just a multiple of that vector of effects. If only it were that easy!

Yes, I completely see the point here. I did much reading following this comment (some choice items on this list are now newly cited), and I fear R3 is right. Resisting the temptation to (unpersuasively) play economist, I decided that doing justice to this analysis is beyond my physical science expertise. As I now explicitly openly own up, this will have to follow my work, hopefully by actual economists. Given that this is *PLOS Biology*, however, I feel that the current paper stands without this

For example, I could ask, from a UK perspective, what is the national environmental effect if all farms recycled manure, grew vegetables, replaced orchards, grew cover crops, reduced nitrogen use per hectare on wheat to circa 100kg N per ha (this is low for the UK) … ? We do know very clearly that producing less ruminant-based livestock (beef and sheep in particular) and eating more plants will have quite large beneficial effects; personally I think the GHG effects are over-emphasised and nutrient-cycling benefits are under-emphasised: agriculture is still a relatively small emitter of GHGs

Strongly agreed, as most of my papers of the last 5-7 years repeatedly emphasize.

in western economies and the potential negative trade-offs of even small reductions in food supply are high, both in terms of probability of effect and level of effect. However, nutrient loss through leaching of nitrogen and run-off of phosphorous are major land-based issues and, as the author shows, correlated with GHG emissions. Thus, my major criticism of the aggregate NSA scenario is that at best it is not sufficiently interesting and at worst it could lead policy makers in a very dangerous direction. While it is stated that an economic analysis is not part of the work I do think that there should be greater acknowledgement that there is an underlying system that cannot just be swapped for the modelled system without substantial disruption. In essence 'industrial' - to use the author's term - agriculture has delivered cheap food that has freed society to do things other than growing food and, as importantly, freed society from worrying about where food will come from
each week. Yes, the environmental cost of this is large and needs addressing; however, I think it is dangerous just to present an end-result without considering the transition to that result. Indeed, a novel paper would address how we move to a better place whilst also addressing the effects of this transition on e.g. consumer food prices.

These are clearly good points. I fear, however, that they are beyond the scope of the current paper or my scientific skills, I am a geophysicist, not an economist. In the spirit of what I wrote earlier regarding scientific follow-ups, I’d love to see this very issue taken up by bone fide economists, and their results may well require subsequent revisions of the current paper’s conclusions. That would be both excellent and fascinating! This is what I added to the ms to address this well-argued point. “The economic and broader societal dimensions of up-scaling the single NSA farm results in scope are far more challenging [50,51]. If embarked on cavalierly or abruptly, the transition from the current state to the full envisioned NSA system may well lead to supply, demand and price shocks, as well as dietary and culinary ones, that most will deem intolerable. Avoiding those shocks and rendering the transition successful thus poses important social science challenges. While they are outside of the current geophysical scope, it is my sincere hope that economists and fellow social scientists will address them in future contributions.” Since this is consistent with R3’s “a novel paper would address “, I hope and trust R3 will recognize the limits of my expertise and the importance of the geophysical focus and the need for this analysis to be performed by others in future contributions.

Regarding the analysis itself, I am uncomfortable with the format for the given topic. While 'Results' followed by 'Methods' is standard for Plos Biology it does make it hard for the reader. For example, Monte Carlo Analysis is mentioned without any explanation of what it is or why it is used. There is a lack of transparency about how nutrient loss is calculated - equations are mentioned but we have to take it on trust that the results are valid. Furthermore, interventions in agriculture designed to improve environmental outcomes are inherently variable: they work, they don't work, they work but then it rains, they don't work but then it rains and they do work… and so forth. 
This isn't very helpful and as I wrote at the start, I do think there is important work to be done here. I would like to see the workings behind the models, some sensitivity analysis - just generally to understand better how the proposed NSA system works on farm and for the environment (one of the questions I am asked as a reviewer is: "Are details of the methodology sufficient to allow the experiments to be reproduced?" - to which I would have to answer 'no'). Perhaps this important work would be more suited to a different type of journal.

Let me address the last point first. I ran the ms by the *PLOS Biology* Editors prior to initial submission, and after examining it preliminarily, they gave me the thumbs up. I like the Journal very much, and think it’s an excellent home for this paper. While the paper has changed since then, its essence has not, and the Editors’ judgement thus seems to me to still hold. Whether or not it will ultimately be published in *PLOS Biology* is of course the Editors’ decision. But I know where I stand: if given the opportunity by the Editors, *PLOS Biology* it is for this paper.

Re the section order, if the Editors OK the non-traditional order swap, I am happy to make it. Within my jurisdiction, following this comment, I added to the Methods section *of the main text* the governing equations and their development into a soluble system. I then also added a brief description of the solution approach, so that the Monte Carlo formalism will be clearer to the reader, as requeste by the Reviewers. Beyond this, I still see the SI as an extremely important and integral part of the paper; one needs all the details in the SI to reproduce the results and understand the paper, but one definitely does *not* want them interrupting the narrative flow in the main text. I guess relegating technical details to the SI is the trend in scientific publishing. I personally like it to some extent⎯if we want to quickly get the general thrust and bottom line of a particular paper, this format is excellent⎯but also find it sometimes frustrating, which I think is exactly the sentiment R3 voices here. I picture most readers of this paper will download and read it as a single combined PDF, and will follow the narrative by alternating between the main text and the SI as needed. So the “workings behind the model” are indeed readily available to the reader, following the above revisions now more so than earlier, as R3 favors, split between the SI and the main text. Finally, note that there are now extensive sensitivity analyses to various parameterized part of the model (again, in the SI) and they clearly indicate robustness of the results under uncertainty of those parametrizations.

COMMENTS FROM THE ACADEMIC EDITOR [lightly edited]:

I agree with your way forward [Major Revision] but equally share your [my!] concern about something coming back half-baked. Reviewer #2 is quite spot-on for me that it's not the easiest paper to read.

So how to lend some more direction? Technically, there are I think quite serious concerns about the appropriateness of the N use efficiencies raised by Rev #2 that should be explored with further analysis. This is where the sensitivity analyses from Rev #3 could partly come in.

Done as prescribed.

Rev #3 also raises a major concern about the scaling of the system being linear (a "vector") and whether that is appropriate. Again, do we need further (sensitivity?) analysis here?

Please see my response directly to R3. This is a fair point in general, but is not an essential element of the current paper. Please see my detailed specific response above.

Beyond this modelling details, both Revs #2 and #3 lament the way the workings of the modelling and underlying equations / assumptions are presented. More detail and precision is clearly needed in a revision and that should be a straightforward point to communicate to the author.

As I said earlier, I’m OK with the SI being an essential element of the paper. I survey of recent *PLOS Biology* papers suggests that far from unique, this is in fact customary in this journal.

Finally, I think the points raised by Rev #3 about the underlying economics are important to acknowledge in a meaningful way. Less sure about how this one looks, but maybe a discussion of the limitations of the current approach w.r.t. economics might be sufficient?

Thanks for this, a good synopsis of the reviews. As I said earlier, following the universal agreement of Reviewers and Editor alike, I rewrote much of the narrative, trying my best to enhance readability and thread. I also now address head on the limitations posed by the absence of an economic analysis, which I beyond the current scope.

**Thanks much, one last time, to all involved, it was a pleasure getting your feedback!**

1. [*Carter et al., Holistic Management: Misinformation on the Science of Grazed Ecosystems*](https://downloads.hindawi.com/archive/2014/163431.pdf)*, International Journal of Biodiversity* Vol. **2014** #163431 doi: 10.1155/2014/163431.
2. *Rangelands* **35**(5):72–74, doi: 10.2111/RANGELANDS-D-13-00044.1.
3. SLU/EPOK – Centre for Organic Food & Farming & Chalmers, ISBN 978-91-576-9424-9, Swedish University of Agricultural Sciences & Chalmers, Uppsala.
