# Supplementary material for: Small-scale integrated farming systems can abate continental-scale nutrient leakage
Source: PLoS Biol. 2021 Jun 3;19(6):e3001264. doi: 10.1371/journal.pbio.3001264 (PMC8174726; doi:10.1371/journal.pbio.3001264)
Supplement: Attachment — Submitted filename: response2reviewsV3.docx [file pbio.3001264.s006.docx]

Gidon Eshel

43 Wynkoop Ln.

Rhinebeck

NY 12572

(413) 717-2187

[geshel@gmail.com](mailto:geshel@gmail.com)

Tuesday, April 27, 2021

Dr. Roland G. Roberts, Senior Editor

*PLOS Biology*

Dear Editor Roberts,

Thank you for handling the paper (now PBIOLOGY-D-20-02894R2) and for your decision email. Much appreciated. Please see my responses below.

Thanks so much again and all the best, Gidon

**Editor notes:**

Re the title: yeah, that makes sense. I changed it to almost verbatim what Editor Roberts proposed, plus epsilon I favored: continent*al* instead of continent, and retaining the leakage, because losses emphasizes fertilizer costs, which I do *not* address, while leakage emphasizes eutrophication, which is the real rationale here.

Fig. 3e is now sporting its previously missing e.

All Figures are updated (because I had to rerun all randomizations) and the captions are appended, as instructed, to indicate that numerical values are available in a Supplementary data file I have now generated and that accompanies this submission.

**Reviewer 2:**

1. Done.
2. Abstract edited in accordance with this nice point.
3. The par in question is in fact not in my original submission, but was added in round 1 of the review process to address a specific request for clarity by one of the initial Reviewers. While Rev. 2 is obviously right, the fact that one of the reviewers was not clear on that and requested clarification, claiming that C sequestration is entirely distinct from eutrophication reduction, suggests others might be similarly mistaken. That said, I rewrote extensively the par in question and the one following in an effort to add clarity and conform with Reviewer 2’s current objection.
4. This addresses that following par mentioned above, and I do believe that "… also partly controls sequestration…" now clearly follows.
5. Reviewer 2 is dead on right here, my bad. I reworded the sloppy sentence to be, hopefully, clear and correct now.
6. Par shortened and rewritten (now around line 150). It was indeed a tad wordy, and is less so now.
7. Fixed (now at the end of the par around line 187).
8. I reworded somewhat, but this again was added based on an earlier Reviewer comment. Even though I didn’t write it initially, I like it, I think it’s fairer to the reader to be upfront about these limitations. Apart from the afore-mentioned rewording, I thus left the caveats in place, but made them somewhat shorter.
9. Re exports, I was only able to find any information is financial terms, not in terms of land use. There appears to be genuinely no such estimate. I don’t think it’s very illuminating for this paper. Here is why: some 75% of total exports is meat and feed^[1]^. Which is to say, exports are *mostly* about things that will have to change radically. The comparison, just quoting so many acres for export, is therefore not only impossible (as indicated above) but also minimally meaningful.
10. I think it’s entirely obvious: while the earlier paper was written by several authors, here I am traveling solo. I added a clarification (around line257) about the rangeland, it follows logically with no issues that I can discern.
11. Thank you, good catch, fixed (around line 300).
12. This point was indeed confusing and not essential. I thus got rid of the end of the offending par.
13. I added a parenthetical definition of vegetal the first two times it appears (because it is once in a Figure caption and once in the main text. It is a short word replacing a long sentence, the word is nice and legit, and it describes exactly what I intended.

**Thanks much, one last time, to all involved, it was a pleasure getting your feedback!**

1. https://www.thebalance.com/us-agriculture-stats-facts-history-4776144
